# Vaccinia-related kinase 2 inhibition elicits vulnerability of glutathione metabolism in pancreatic cancer

**DOI:** 10.1038/s41419-026-08573-9

**Published:** 2026-03-19

**Authors:** Sisi Chen, Xiaowei Fu, Tianyue Zhang, Rui Zhou, Long Liu, Liuhai Zeng, Bin Xu, Hengqing Zhu, Zhiyu Li

**Affiliations:** 1 Department of Neurology, Second Affiliated Hospital of Nanchang University, Nanchang, 330006 Jiangxi P. R. China; 2 Department of Hepatobiliary and Pancreatic Surgery, Second Affiliated Hospital of Nanchang University, Nanchang, 330006 Jiangxi P. R. China; 3 Department of Endocrinology, Second Affiliated Hospital School of Medicine, Zhejiang University, Hangzhou, 310009 Zhejiang P. R. China; 4 Department of Hepatobiliary and Pancreatic Surgery, Second Affiliated Hospital School of Medicine, Zhejiang University, Hangzhou, 310009 Zhejiang P. R. China; 5 Xiangshan First People’s Hospital Medical and Health Group, Ningbo, 315700 Zhejiang P. R. China; 6 Department of Burns, First Affiliated Hospital of Nanchang University, Nanchang, 330006 Jiangxi P. R. China; 7 Department of Thyroid Surgery, Second Affiliated Hospital School of Medicine, Zhejiang University, Hangzhou, 310009 Zhejiang P. R. China; 8 Center for Medical Research and Innovation in Digestive System Tumors, Minstry of Education, Hangzhou, 310009 Zhejiang P. R. China

**Keywords:** Cancer metabolism, Tumour biomarkers

## Abstract

Metabolic reprogramming has garnered significant attention in recent years due to its therapeutic potential in cancer treatment. However, identifying responsive tumor subpopulations remains a major obstacle in developing metabolism-targeted therapies, as metabolic vulnerabilities vary among cancers with different oncogene expression profiles. Therefore, elucidating the association between oncogene expression and metabolic characteristics could enable more precise metabolic interventions in clinical settings. Using pharmacological approaches, we demonstrate that VRK2-deficient pancreatic cancer (PC) cells exhibit heightened vulnerability to glutathione (GSH) metabolic pathway inhibition. This susceptibility stems from reduced basal GSH levels caused by impaired plasma membrane expression of SLC7A11. Mechanistically, we reveal that VRK2 inhibition disrupts endoplasmic reticulum (ER)-to-Golgi trafficking of SLC7A11, consequently diminishing GSH biosynthesis and predisposing PC cells to ferroptosis. Collectively, our findings establish a novel link between the oncogene VRK2 and GSH synthesis metabolism, providing a molecular basis for developing stratified metabolic therapies for PC patients.

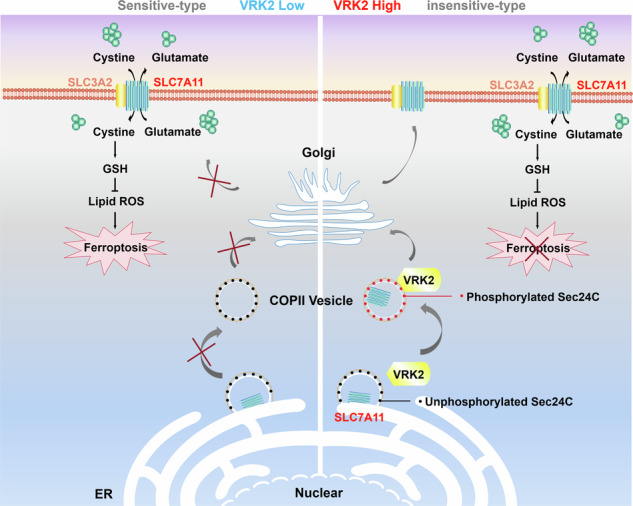

## Introduction

Pancreatic cancer(PC) is one of the most lethal malignancies with a 5-year survival rate less than 9% [1]. Current epidemiological data rank it as the seventh leading cause of cancer-related mortality worldwide, with projections indicating it may become the second leading cause in the United States by 2030 [2, 3]. Although substantial efforts have been made to improve clinical outcomes, existing therapeutic strategies demonstrate limited efficacy. This therapeutic impasse underscores the urgent need for novel breakthroughs in PC treatment. In recent years, increasing attraction has been paid to metabolic network as for cancer cells have the capability to support survival and growth by adopting extensive metabolic rewiring in response to alterations in internal or external circumstances [4, 5]. Thus, targeting the metabolic reprogramming may provide appealing opportunities to explore novel treatments for PC.

Oxidative stress caused by high levels of reactive oxygen species(ROS) can lead to cell damage and death. Intracellular ROS levels depend on the balance between ROS generation and clearance, and are also manipulated by antioxidant defense mechanisms [6]. Glutathione (GSH), the most potent endogenous antioxidant identified to date, safeguards cells by participating in redox reactions that neutralize accumulated ROS [7]. Of note, GSH serves as the terminal product of the GSH synthesis pathway, which utilizes cysteine, glutamate, and glycine as primary substrates. This biosynthetic process is mediated by glutamate-cysteine ligase (GCL), which includes glutamate-cysteine ligase catalytic subunit(GCLC) and GSH synthetase(GSS) [8]. Cysteine is a rate-limiting precursor substrate for GSH synthesis, and its level is regulated by SLC7A11 encoding the cystine/glutamate transporter XCT, which is primarily responsible for the export of glutamine in exchange for extracellular cystine [9]. Therefore, in order to overcome the survival crisis caused by oxidative stress, cancer cells are likely to exhibit metabolic preferences and undergo metabolic reprogramming, including but not limited to GSH metabolic rewiring.

Evidence have shown that the process of metabolic reprogramming can be regulated by the underlying genetic factors that drive tumorigenesis. For example, MYC and KRAS overexpression are involved in diverse metabolic changes, including increased glucose uptake and rewiring of GSH biosynthesis [10, 11]. The oncogenic transcription factor c-Jun is reported to elevate the expression of mitochondrial glutaminase by directly binding to its promoter region in breast cancer cells [12]. However, it remains unclear whether PC cells with certain carcinogenic activity exhibit a specific metabolic preference among numerous metabolic changes.

Vaccinia-related kinase(VRK) is a newly discovered serine-threonine kinase in humans. VRK2 is one of the most characteristic members of the VRK family. It is located on human chromosome 2p16, and the protein encoded by the VRK2 gene is expressed in the human brain, testis, thymus and fetal liver [13]. To date, the aberrant expression of VRK2 protein has been proven to play a role in the development of various tumors [14–16]. Our previous studies have illustrated that VRK2 is an oncogenic driver of malignant growth of PC, and its overexpression is positively correlated with poor outcome of PC [17]. A recent investigation revealed that the expression of VRK2 was upregulated in response to ROS signals, while the link between VRK2 and metabolic rewiring remains obscure [18].In this study, we tried to explore the effects of VRK2 on the rewiring of metabolic networks and identify metabolic vulnerabilities resulting from VRK2-driven metabolic reprogramming. Here, we found that VRK2-deficient PC cells exhibited increased GSH dependency and were susceptible to the inhibition of the GSH metabolic pathway. Mechanistically, oncogenic VRK2 activation induced GSH synthesis by promoting vesicle trafficking and membranal expression of SLC7A11, and consequently supported survival of PC cells. Our findings provide a basis for stratifying VRK2-aberrant patients for metabolism-targeted therapies.

## Materials and methods

### Cell culture

The human pancreatic cancer cell lines (CFPAC-1, SW1990, PANC-1, and BxPC-3) and murine pancreatic cancer cell line (Pan02) were procured from the Cell Bank of the Chinese Academy of Sciences Type Culture Collection (Shanghai, China). Human-derived cells were cultured in DMEM medium (Gibco, Grand Island, NY, USA) supplemented with 10% fetal bovine serum (FBS; Gibco), while murine Pan02 cells were maintained in RPMI-1640 medium (Gibco) with 10% FBS. All cell lines were incubated at 37 °C in a humidified atmosphere containing 5% CO₂.

### Cell transfection

The shVRK2-expressing and VRK2-overexpressing plasmids were purchased from Genechem (Shanghai, China) and then used for constitutive expression of shRNA or cDNAs. CFPAC-1 and SW1990 cell lines were transfected with shVRK2 plasmids, and PANC-1 and BxPC-3 cell lines were transfected with VRK2-overexpressing plasmids by applying Lipofectamine 3000 reagent according to the manufacturer’s protocol. Stable expression of shRNA or cDNAs were screened for 4 weeks with puromycin (2.0 mg/mL). SLC7A11-, GCLC-, and GSS-overexpressing plasmids were purchased from Origene (USA). The siRNA sequences targeting SLC7A11, GCLC, and GSS were synthesized by Genepharma (Suzhou, China). Transfection of overexpressing or siRNA plasmids were carried out in 60–70% confluent cells by using Lipofectamine 3000 reagent according to the manufacturer’s protocol.

### Measurement of GSH and GSSG levels

For each cell line, a total of 4000 cells/well were seeded into 96-well plates. After 24 h of continuous culture, the media was discarded and displaced by media containing agents with specified concentrations as indicated. The cells were then washed with PBS, and GSH and GSSG levels were determined by applying the GSH/GSSG-Glo assay according to manufacturer instructions.

### Detection of ROS

Cells were seeded in 6-well plates and treated with the indicated agents, followed by 10 mM H2DCFDA when cells grew to 70% confluence. The cells were washed twice with PBS, trypsinized, and then stained with propidium iodide(1 mg/mL). H2DCFDA fluorescence (FL-1) in propidium iodide-negative cells (FL-3) was tested by a FACSCalibur Flow Cytometer.

### Transmission electron microscopy (TEM)

TEM assays were performed as previously described [14]. PC cells were harvested after trypsinization, followed by centrifugation and then fixed at 4 °C overnight in Karnovsky’s fixative (2% paraformaldehyde and 5% glutaraldehyde in 0.1 M cacodylate, pH 7.4). Post-fixation processing was performed in 1% osmium tetroxide for 2 h. The cells were dehydrated in a graded series of alcohol and acetone and embedded in Araldite (Spi-Chem, USA). Ultrathin sections were obtained by using a Leica ultramicrotome (Leica Microsystems, Buffalo Grove, IL, USA) and double-stained with uranyl acetate and lead citrate (Spi-Chem, USA). TEM images were observed under a TEM (JEOL1230, Japan).

### Animal experiments

All animal procedures were performed in accordance with the ethical standards approved by the Animal Care and Ethics Committee of the Second Affiliated Hospital, Zhejiang University School of Medicine. For the orthotopic pancreatic cancer model, VRK2-WT or VRK2-KO Pan02 cells in logarithmic phase (80–90% confluence) were harvested, detached with 0.25% trypsin-EDTA, centrifuged (1000 rpm, 5 min), and resuspended in cold serum-free RPMI-1640 medium. The cell suspension was adjusted to the desired density, mixed with ice-cold Matrigel, and kept on ice to prevent gelation. Male C57BL/6 mice (6–8 weeks old) were randomly divided into different subgroups and anesthetized with 1.5–3% isoflurane, and a left abdominal incision was made to expose the pancreas. A 100 μL cell-Matrigel suspension was injected into the pancreas using a 27-gauge needle, followed by closure of the peritoneum with absorbable sutures and the skin with wound clips. Mice were monitored daily for 7 days post-surgery for distress. On day 10 after inoculation, tumor-bearing mice were treated with vehicle (saline), APR-017(100 mg/kg per day, ip., for 10 days), IC261(0.6 mg/kg per day, ip., for 10 days), or APR-017 + IC261, and tumor growth was monitored via in vivo imaging (IVIS). For subcutaneous xenografts, BALB/c-nu/nu mice (male, 6–8 weeks, 18 ± 1.5 g) were housed under controlled conditions (22–24 °C, 40–60% humidity, 12-h light cycle) and randomly divided into subgroups. Following anesthesia (1.5–3% isoflurane, Sigma-Aldrich), 1 × 10⁶ PDCs in 100 μL PBS were injected into the unilateral flank. Treatment began when tumors reached ~100 mm³, with mice randomized to vehicle, APR-017(100 mg/kg per day, ip. for 10 days) or vehicle, APR-017(100 mg/kg per day, ip., for 10 days), IC261(0.6 mg/kg per day, ip., for 10 days), combination therapy. Tumor volume (V = ½ × length × width²) was measured every 5 days until endpoint, when IVIS imaging preceded final mass assessment.

For further details regarding the methods used, please refer to the Supplementary Materials and Methods.

### Statistical analysis

The results are presented as mean ± SD from at least three independent biological replicates, with all analyses performed using GraphPad Prism 7 (GraphPad Software, USA). For statistical comparisons, two-tailed Student’s t-tests were employed for two-group comparisons, while one-way or two-way ANOVA with Bonferroni correction was used for comparisons involving three or more groups. Statistical significance was established at *P* < 0.05. All in vitro experiments were biologically replicated at least three times, and the specific numbers of animals used in each experiment are detailed in the corresponding figure legends.

## Results

### VRK2-deficient PC cells are vulnerable to GSH inhibition

**Fig. 1:**
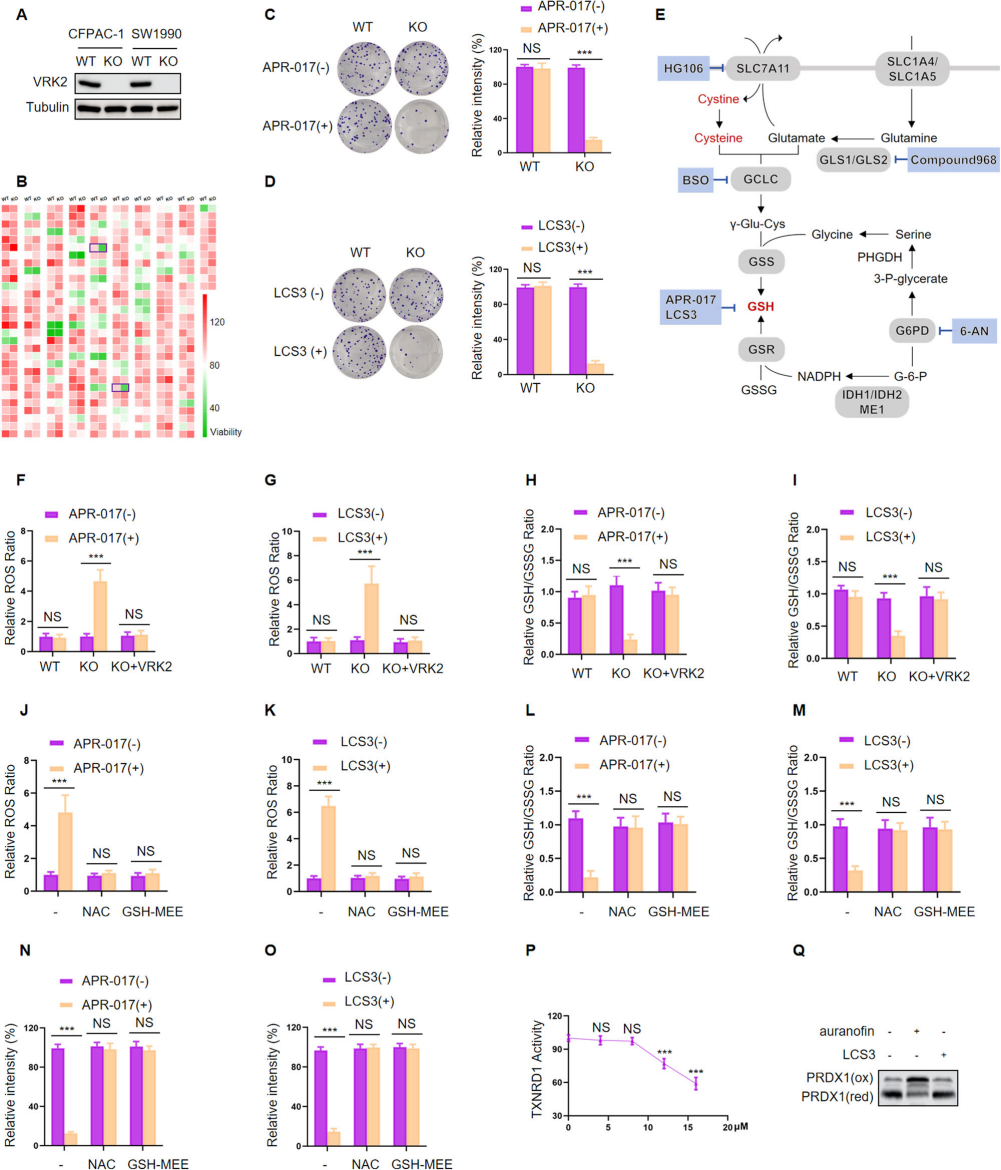
VRK2-deficient PC cells are sensitive to GSH inhibition. **A** VRK2 knockout in PC cells was validated by western blot; **B** Heatmap showing the cell viability of VRK2-WT and VRK2-KO PC cells after treatment with 281 compounds (each at 10 μM) for 3 days. The boxes indicated the viability of PC cells treated with APR-017 or LCS3. **C**, **D** VRK2-WT and VRK2-KO PC cells were exposed to APR-017 (10 μM, 72 h) or LCS3 (10 μM, 72 h) and then cultured for 14 days. Cell viability was assessed by colony formation assay. *n* = 3; NS no significant. ***p < 0.001. **E** Schematic showing the metabolic products and inhibitors of enzymes involved in the GSH metabolic pathway. **F**–**I** Relative ROS levels (**F**, **G**) and relative GSH levels (**H**, **I**) were examined in VRK2-WT, VRK2-KO, and VRK2 restoring VRK2-KO cells after treatment with 25 μM APR-017 or 15 μM LCS3 for 48, 48, 24, 24 h, respectively. *n* = 3; NS no significant. ***p < 0.001. **J**–**O** Relative ROS levels (**J**, **K**), relative GSH levels (**L**, **M**) and cell viability (**N**, **O**) were tested in VRK2-KO cells after treatment with APR-017 (25 μM) or LCS3 (15 μM) for 48, 48, 24, 24, 72, 72 h, respectively, without or with co-treatment of NAC (10 mM) or GSH-MEE (5 mM). *n* = 3; NS no significant. ***p < 0.001. **P** Relative TrxR activities were detected in PC cells with different concentrations of LCS3 treatment for 24 h. *n* = 3; NS no significant. ***p < 0.001. **Q** Redox western blot showing PRDX1 redox status in VRK2-KO cells treated with auranofin (5 μM) or LCS3 (10 μM).

To investigate the impact of VRK2 on metabolic reprogramming, we treated VRK2-wild-type (VRK2-WT) and VRK2-knockout (VRK2-KO) CFPAC-1 cell lines with 281 metabolism compounds (Supplementary Table1). A drug-sensitivity analysis showed that the cellular survival rate of VRK2-KO cells was significantly lower than that of VRK2-WT cells when treated with APR-017 or LCS3 (Fig. 1A, B). To verify this result, we assessed the selective sensitivity of VRK2-KO cells (CFPAC-1 cells and SW1990 cells) to APR-017 or LCS3 by employing colony formation assays. We observed that both APR-017 and LCS3 reduced the viability of VRK2-KO cells, but not VRK2-WT cells (Fig. 1C, D and Supplementary Fig. 1A, B). Given that both APR-017 and LCS3 are involved in inhibiting the GSH synthesis metabolism pathway, we examined whether the lethal effects of APR-017 and LCS3 on VRK2-deficient cells were due to inhibition of GSH. We found that APR-017 or LCS3 treatment induced an increased level of ROS and a decreased level of GSH (presented as the ratio of GSH to oxidized form, GSSG) in VRK2-KO cells, but not VRK2-WT cells (Fig. 1E–I and Supplementary Fig. 1C–F). Interestingly, the negative regulatory effect of APR-017 and LCS3 on GSH synthesis in VRK2-KO cells was reversed by VRK2 rescuing (Fig. 1F–I and Supplementary Fig. 1C–F). Furthermore, the data showed that both the ROS scavenger *N*-acetylcysteine (NAC) and GSH compensator GSH-MEE could abrogate APR-017 and LCS3 induced increase in ROS, decrease in GSH and inhibition of cell growth in VRK2-KO cells (Fig. 1J–O and Supplementary Fig. 1G–L), indicating that GSH inhibition is a key factor promoting the sensitivity of VRK2-deficient cells to APR-017 and LCS3.

In addition to GSH inhibition, LCS3 is reported to inhibit the activity of thioredoxin reductase1 (TXNRD1), a critical antioxidant regulator maintaining the redox state of thioredoxin (TRX) [19](Supplementary Fig. 1M). Hence, we determined if the decreased cellular viability and increased ROS caused by LCS3 in VRK2-deficient cells could be partly explained by TXNRD1 inhibition. The data showed that the same concentration of LCS3 mentioned above did not affect the activity of TXNRD1 in VRK2-KO cells, but a higher concentration of LCS3 was required to induce a decline in the activity of TXNRD1 (Fig. 1P and Supplementary Fig. 1N). Additionally, the oxidation status of peroxiredoxin 1 (PRDX1), a direct substrate of TRX, was tested by employing a redox western blot to reflect the activity of TXNRD1 indirectly. Auranofin, a specific inhibitor of TXNRD1, was used as a positive control. In comparison with markedly increased oxidation of PRDX1 induced by auranofin, no significant increase was observed in VRK2-KO cells treated with the above concentration of LCS3 (Fig. 1Q and Supplementary Fig. 1O), implying that the lethality of VRK2-deficient PC cells induced by LCS3 scarcely depends on TXNRD1 inhibition. Collectively, these results suggested that APR-017 and LCS3 treatment induce vulnerability of VRK2-deficient PC cells by GSH inhibition and consequent disruption of the balance between ROS generation and antioxidation.

### SLC7A11 is a critical regulatory target involved in VRK2 regulating the GSH metabolic synthesis pathway

**Fig. 2:**
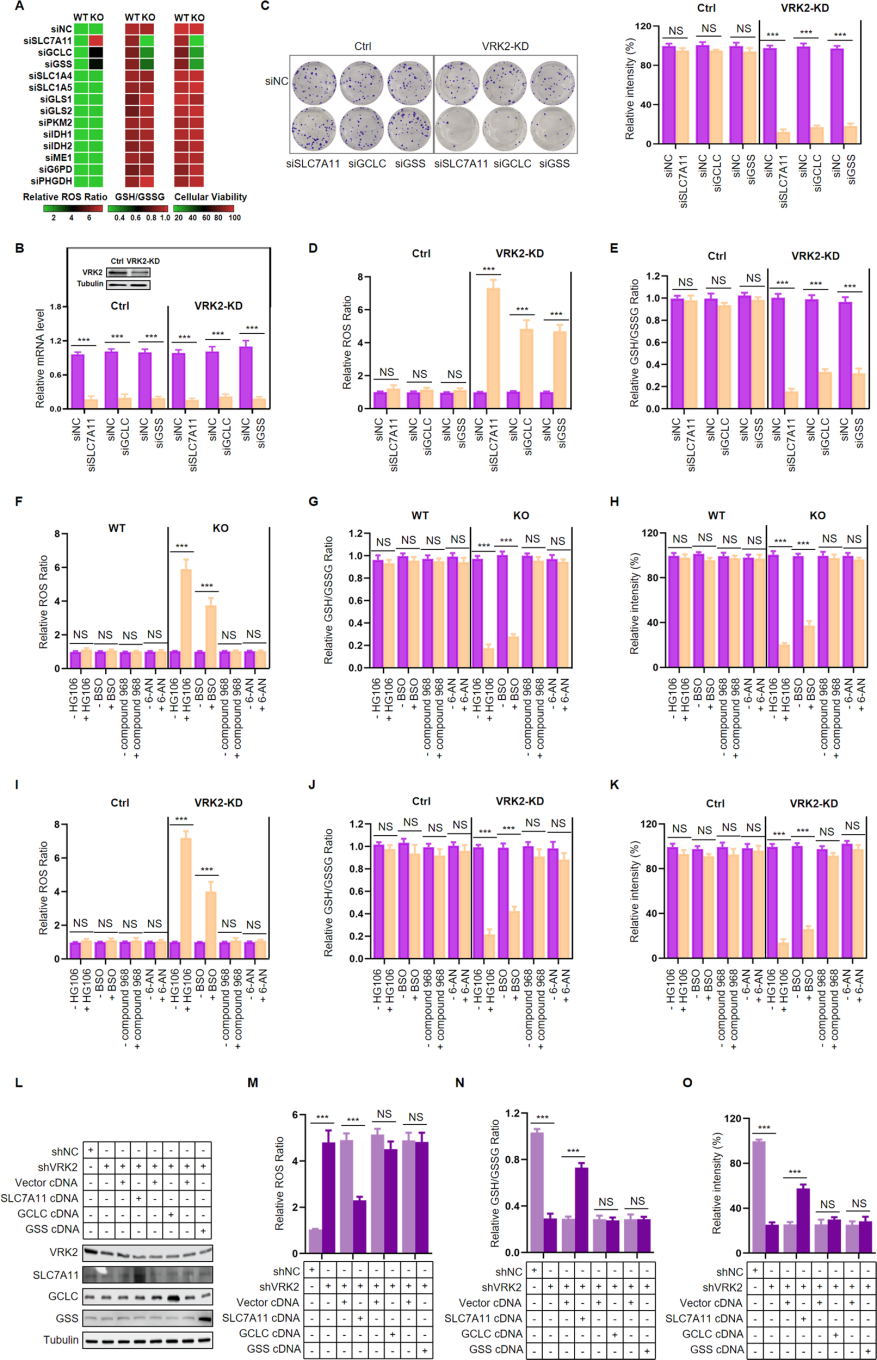
SLC7A11 is a key target involved in VRK2 rewiring GSH metabolism. **A** Heatmap showing relative ROS levels, GSH levels and cell viability in VRK2-WT and VRK2 KO cells at 48, 24, and 48 h, after knockdown of GSH pathway genes. **B** VRK2 knockdown in PC cells was validated by western blot (upper panel), and the downregulation of SLC7A11, GCLC, and GSS were confirmed by RT-PCR (lower panel). *n* = 3; ***p < 0.001. **C** Viability of shNC and shVRK2-transfected PC cells with SLC7A11, GCLC, and GSS knockdown, respectively. *n* = 3; NS no significant. ***p < 0.001. **D**, **E** Relative ROS levels (**D**) and relative GSH levels (**E**) were examined in shNC and shVRK2-transfected PC cells with SLC7A11, GCLC, and GSS knockdown, respectively. *n* = 3; NS no significant. ***p < 0.001. **F**–**H** Relative ROS levels (**F**), relative GSH levels (**G**), and viability (**H**) were assessed in VRK2-WT and VRK2 KO cells after treatment of HG106 (1 μM), BSO (10 μM), compound 968 (6 μM), and 6-AN (10 μM) for 48, 24, and 48 h, respectively. *n* = 3; NS no significant. ***p < 0.001. **I**–**K** Relative ROS levels (**I**), relative GSH levels (**J**), and viability (**K**) were assessed in shNC and shVRK2-transfected PC cells after treatment of HG106 (2 μM), BSO (15 μM), compound 968 (10 μM), and 6-AN (15 μM) for 48, 24, 48 h, respectively. *n* = 3; NS no significant. ***p < 0.001. **L** SLC7A11, GCLC and GSS overexpression was validated by western blot in shNC and shVRK2-transfected PC cells. **M**–**O** Relative ROS levels (**M**), relative GSH levels (**N**), and viability (**O**) were evaluated in shNC and shVRK2-transfected PC cells at 48, 24, and 48 h, after upregulating the expression of SLC7A11, GCLC, or GSS, respectively. *n* = 3; NS no significant. ***p < 0.001.

Aforementioned data indicated that VRK2-deficient PC cells are vulnerable to GSH inhibition, this prompts us to explore the link between VRK2 and GSH metabolic synthesis pathway. We downregulated the expression of genes related to the GSH metabolic pathway in VRK2-KO PC cells and examined their effects on cellular viability, ROS and GSH. As shown in Fig. 2A and Supplementary Fig. 2A, compared with VRK2-KO cells downregulating the paralog genes involved in the GSH metabolic pathway, VRK2-KO cells with GCLC, GSS, and especially SLC7A11 knockdown exhibited increased ROS level, decreased GSH level and cellular viability. Similar results were observed in VRK2-knockdown(KD) PC cells (Fig. 2B–E and Supplementary Fig. 2B–E). In addition, small molecule inhibitors including SLC7A11 inhibitor HG106 and GCLC inhibitor buthionine sulfoximine (BSO) were introduced to investigate their effects on ROS, GSH levels and cellular viability in VRK2-KO and VRK2-KD cells, and the results were consistent with the above knockdown experiment (Fig. 2F–K and Supplementary Fig. 2F–K). Nevertheless, other inhibitors, including GLS1/GLS2 inhibitor compound 968 and G6PD inhibitor 6-aminonicotinamide (6-AN) were failed to induce the above alterations in VRK2-KO and VRK2-KD cells (Fig. 2F–K and Supplementary Fig. 2F–K). These results imply that GCLC, GSS and SLC7A11 are the potential regulatory targets involved in the VRK2-driven metabolic synthesis pathway of GSH.

We next determined if VRK2 played a role in the GSH metabolic synthesis pathway by regulating one or more of GCLC, GSS, and SLC7A11. We upregulated the expression of GCLC, GSS, or SLC7A11 in VRK2-KD PC cells, and investigated the effects of these genes on cell viability, ROS and GSH levels when provided exogenous ROS ter-tbutyl hydroperoxide (TBHP). The results showed that VRK2 knockdown could induce an increase of ROS and decline of cell viability and GSH following TBHP treatment, while these alterations could be partly reversed by ectopic SLC7A11 expression but not GCLC or GSS expression (Fig. 2L–O and Supplementary Fig. 2L–O). Therefore, these results indicate that SLC7A11 is a critical regulatory target involved in VRK2 regulating the GSH metabolic synthesis pathway.

### VRK2 promotes ER-to-Golgi trafficking of SLC7A11 and its expression on the cell membrane

**Fig. 3:**
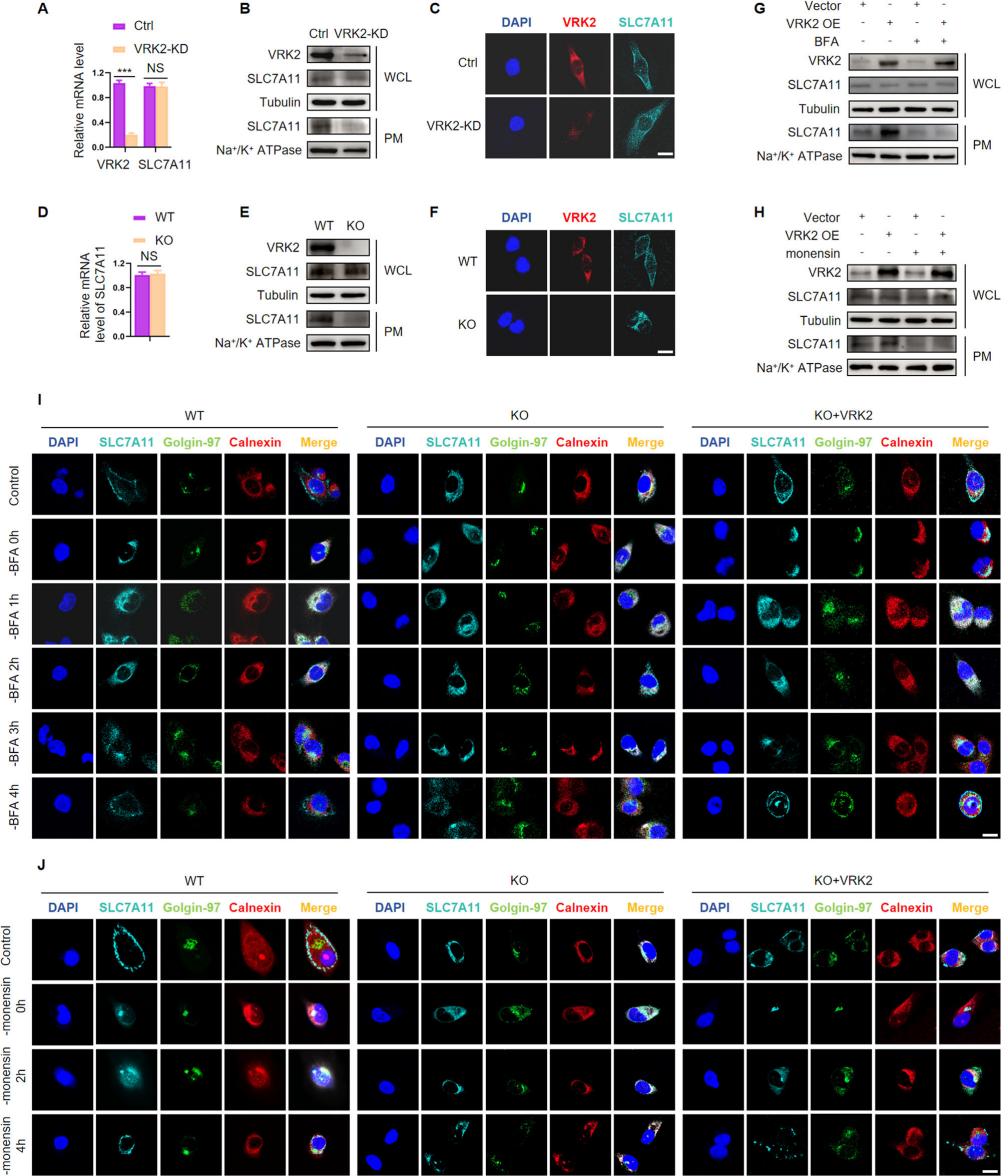
VRK2 promotes ER-to-Golgi trafficking of SLC7A11 and its expression on the cell membrane. **A** mRNA of VRK2 and SLC7A11 in shNC or shVRK2-transfected PC cells was tested by RT-qPCR. n = 3; NS, no significant. ***p < 0.001. **B** Immunoblot for protein expression of SLC7A11 at the whole cell level and plasma membrane level in PC cells expressing shNC or shVRK2; WCL, whole cell lysate; PM, plasma membrane. **C** Subcellular location of SLC7A11 in PC cells expressing shNC or shVRK2 was detected by immunofluorescence; Scale bar indicates 20 μm. **D** mRNA of SLC7A11 in VRK2-WT and VRK2-KO PC cells. *n* = 3; NS, no significant. **E** Immunoblot for protein expression of SLC7A11 at whole cell level and plasma membrane level in VRK2-WT and VRK2-KO PC cells; WCL, whole cell lysate; PM, plasma membrane. **F** Subcellular location of SLC7A11 in VRK2-WT and VRK2-KO PC cells was detected by immunofluorescence; Scale bar indicates 20 μm. **G**, **H** Immunoblot for protein expression of SLC7A11 at whole cell level and plasma membrane level in control vector or exogenous VRK2-transfected PC cells treated without or with BFA (10 μg/ml, 1 h) or monensin (3 μM, 6 h). **I** ER-to-Golgi trafficking of SLC7A11 in VRK2-WT, VRK2-KO, and VRK2 restoring VRK2-KO cells. Cells were treated with 10 μg/ml BFA for 1 h and then analyzed by laser scanning confocal microscope at different times after BFA removal. The endoplasmic reticulum was labeled with calnexin antibody. The Golgi apparatus was labeled with the Golgi-97 antibody. Scale bar indicates 20 μm. Scale bar indicates 20 μm. **J** Golgi-to-PM transport of SLC7A11 in VRK2-WT, VRK2-KO, and VRK2 restoring VRK2-KO cells. Cells were treated with 3 μM monensin for 6 h and then analyzed by laser scanning confocal microscope at different times after monensin removal. Scale bar indicates 20 μm.

To explore the effect of VRK2 on the expression of SLC7A11, we first investigated mRNA level of SLC7A11 in VRK2 knockdown cells and found that VRK2 did not affect the transcription of SLC7A11 (Fig. 3A and Supplementary Fig. 3A). Western blot assays showed that no significant alteration in the expression of SLC7A11 was observed in VRK2-downregulating PC cells (Fig. 3B and Supplementary Fig. 3B). Like other transmembrane proteins, SLC7A11 protein is synthesized in endoplasmic reticulum (ER) and subsequently transferred to the Golgi apparatus by vesicular and ultimately functions on the cell membrane [20]. Thus, we examined the expression of SLC7A11 at plasma membrane (PM) in VRK2 knockdown cells. The results of western blot and immunofluorescence showed that knockdown of VRK2 resulted in the decline of SLC7A11 expression at PM (Fig. 3B, C and Supplementary Fig. 3B, C). Similar results were obtained using VRK2-WT and VRK2-KO cells (Fig. 3D–F and Supplementary Fig. 3D–F).

Thus, we speculated that VRK2 had an influence on the intracellular trafficking of SLC7A11. To verify this hypothesis, we upregulated the expression of VRK2 in PC cells (PANC-1 cells and BxPC-3 cells) and examine the expression of SLC7A11 at whole cell and PM levels. VRK2 overexpression induced an increase in the expression of SLC7A11 at the PM level but not at the whole cell level. Brefeldin A (BFA), a ER-to-Golgi transport inhibitor, and monensin, a Golgi-to-PM transport inhibitor, abrogated VRK2 overexpression induced increase in the expression of SLC7A11 at PM level (Fig. 3G, H and Supplementary Fig. 3G, H). Furthermore, we investigated whether VRK2 affects the transport of SLC7A11 by regulating ER-to-Golgi or Golgi-to-PM processes. A model system previously reported was utilized to analyze ER-to-Golgi transport in which the cargo was redistributed to the ER in the presence of BFA and exported to the Golgi upon BFA removal [21]. In VRK2-WT cells, as expected, most of SLC7A11 was redistributed to the ER after 1 h of BFA treatment, while at 4 h after BFA removal, almost all the cells showed co-localization of SLC7A11 and the Golgi marker Golgin-97. In contrast, although BFA treatment induced SLC7A11 to be stuck in the ER, only ~15% of VRK-KO cells returned SLC7A11 to the Golgi 4 h after BFA removal. Restoring the expression of VRK2 in VRK2-deficient cells made SLC7A11 redistribute to Golgi after BFA removal (Fig. 3I and Supplementary Fig. 3I). In addition, we treated PC cells with monensin to make SLC7A11 remain in Golgi and trace its expression upon monensin removal. In VRK2-WT cells, after monensin treatment for 6 h, most of SLC7A11 was redistributed to the Golgi, and then returned to the PM after monensin removal for 4 h. In VRK2-KO cells, most of cells displayed ER distribution of SLC7A11 regardless of monensin addition or removal, suggesting that VRK2 did not have an influence on Golgi-to-PM trafficking of SLC7A11 (Fig. 3J and Supplementary Fig. 3J). Collectively, these data indicated that VRK2 had a positive effect on ER-to-Golgi trafficking of SLC7A11, while VRK2 deficiency impede the transport of SLC7A11 .

### VRK2 drives the trafficking of SLC7A11 by phosphorylation of Sec24C

**Fig. 4:**
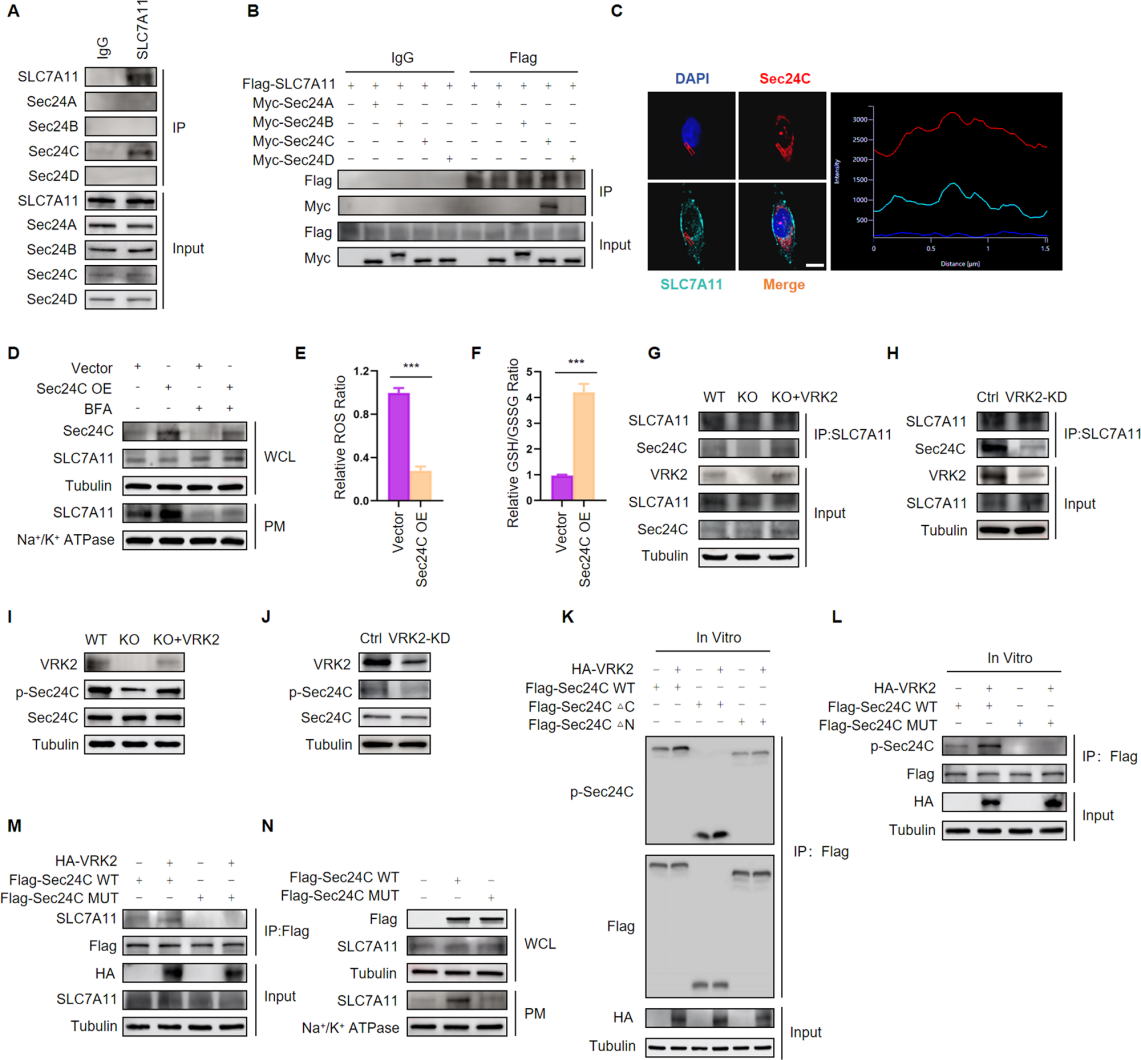
VRK2 promotes the transport of SLC7A11 by phosphorylation of Sec24C. **A** The interaction of endogenous SLC7A11 and Sec24 isoforms were detected by IP. **B** PC cells were transfected with exogenous SLC7A11 and Sec24 isoforms. The interaction of exogenous SLC7A11 and Sec24 isoforms were detected by IP. **C** The co-localization of SLC7A11 and Sec24C was examined by immunofluorescence (left panel). Dimensions result of confocal showed that the intensity of turquoise and red has the same variation tendency(right panel). Scale bar indicates 20 μm. **D** Immunoblot for protein expression of SLC7A11 at whole cell level and plasma membrane level in control vector or exogenous Sec24C-transfected PC cells treated without or with BFA (10 μg/ml, 1 h) or monensin (3 μM, 6 h); WCL whole cell lysate, PM plasma membrane. **E**, **F** Relative ROS levels (**E**) and relative GSH levels (**F**) were evaluated in PC cells transfected with a control vector or exogenous Sec24C. *n* = 3. ***p < 0.001. **G** The interaction of SLC7A11 and Sec24 isoforms in VRK2-WT, VRK2-KO, and VRK2-restoring VRK2-KO cells. **H** The interaction of SLC7A11 and Sec24 isoforms in PC cells expressing shNC or shVRK2. **I** Immunoblot for protein expression of p-Sec24C and total-Sec24C in VRK2-WT, VRK2-KO and VRK2-restoring VRK2-KO cells. **J** Immunoblot for protein expression of p-Sec24C and total-Sec24C in PC cells expressing shNC or shVRK2. **K** VRK2 phosphorylates Sec24C in vitro. Flag-Sec24C WT, Flag-Sec24CΔC or Flag-Sec24CΔN proteins were incubated in vitro with immunoprecipitates isolated from PC cells transfected with constructs encoding HA-VRK2 and then analyzed by western blot using the indicated antibodies. **L** Flag-Sec24C WT or Flag-Sec24C MUT protein was incubated in vitro with immunoprecipitates isolated from PC cells transfected with constructs encoding HA-VRK2 and then analyzed by western blot using the indicated antibodies. **M** PC cells were transfected with HA-VRK2 and Flag-Sec24C WT or Flag-Sec24C MUT. The interaction of SLC7A11 and Flag-Sec24C WT or Flag-Sec24C MUT was analyzed by IP. **N** PC cells were transfected with Flag-Sec24C WT or Flag-Sec24C MUT. The expression of SLC7A11 at the whole cell level and plasma membrane level was then tested by western blot; WCL whole cell lysate, PM plasma membrane.

It is reported that vesicles derived from the ER transporting newly synthesized protein to the Golgi apparatus is driven by Coat protein complex II (COPII) [22]. Sec24 is a critical COPII component responsible for cargo selection in the process of ER-to-Golgi trafficking [23]. In mammals, Sec24 has four isoforms, Sec24 A, Sec24B, Sec24C and Sec24D, which offer the possibility of binding diverse COPII vesicle cargoes [24]. In order to determine which isoform of Sec24 is involved in the sorting of SLC7A11, immunoprecipitation (IP) was carried out to test the interaction of SLC7A11 and Sec24 isoforms. The results of IP showed that SLC7A11 could interact with Sec24C rather than other isoforms (Fig. 4A and Supplementary Fig. 4A), which was also confirmed in PC cells with ectopic expression of SLC7A11 and Sec24 C (Fig. 4B and Supplementary Fig. 4B). Additionally, the co-localization of SLC7A11 and Sec24 C was verified by immunofluorescence assays (Fig. 4C and Supplementary Fig. 4C). Furthermore, we found that Sec24C overexpression elevated the expression of SLC7A11 at PM level, but the effect was abrogated upon exposure to Brefeldin A (Fig. 4D and Supplementary Fig. 4D). Sec24C upregulation also resulted in a decline in the level of ROS and an increase in the level of GSH (Fig. 4E, F and Supplementary Fig. 4E, F). These data suggested that Sec24C interacted with SLC7A11 and promoted its ER-to-Golgi trafficking.

To determine whether VRK2 has an influence on the interaction of SLC7A11 and Sec24C, we checked the affinity of SLC7A11 and Sec24C in VRK2-WT and VRK2-KO cells. We observed that Sec24C displayed a strong interaction with SLC7A11 in VRK2-WT cells but a relatively weak binding affinity toward SLC7A11 in VRK2-KO cells. Restoring the expression of VRK2 in VRK2-KO cells enhanced the interaction of SLC7A11 and Sec24C (Fig. 4G and Supplementary Fig. 4G). VRK2 knockdown also led to reduced binding of Sec24C and SLC7A11 (Fig. 4H and Supplementary Fig. 4H). A previous report demonstrated that phosphorylation exerted a key role in the process of ER-to-Golgi transport regulated by Sec24 [25]. We thus tried to investigate the potential for VRK2 phosphorylating Sec24C and found that the phosphorylation level of Sec24C was attenuated in VRK2-KO cells but elevated when restoring the expression of VRK2 (Fig. 4I and Supplementary Fig. 4I). A decline in the level of phosphorylated-Sec24C was also displayed in VRK2 knockdown PC cells (Fig. 4J and Supplementary Fig. 4J). To investigate whether VRK2 directly phosphorylates Sec24C, we carried out an in vitro kinase assay and found that overexpression of VRK2 was able to increase the phosphorylation level of Sec24C. Besides, we created a truncation of Sec24C and assessed the portion within Sec24C containing potential phosphorylation sites. As the in vitro kinase reaction shown, a truncation of Sec24C consisting of only the first 400 residue (ΔC) rather than the remaining portion (ΔN) was phosphorylated by VRK2 (Fig. 4K and Supplementary Fig. 4K). When serine and threonine residues within the ΔC truncation were replaced by alanine (Sec24C MUT), VRK2 could not increase phosphorylation of Sec24C MUT (Fig. 4L and Supplementary Fig. 4L). VRK2 overexpression elevated the interaction of SLC7A11 and Sec24C WT, but not Sec24C MUT (Fig. 4M and Supplementary Fig. 4M), implying that VRK2 phosphorylated-Sec24C and thereby promoted the interaction of Sec24C and SLC7A11. Furthermore, the expression of SLC7A11 at the PM level was promoted by upregulating Sec24C WT rather than Sec24C MUT (Fig. 4N and Supplementary Fig. 4N). Hence, these results indicated that VRK2 drove the ER-to-Golgi trafficking of SLC7A11 through phosphorylating Sec24C.

### VRK2 protects PC cells from ferroptosis by facilitating SLC7A11 trafficking

**Fig. 5:**
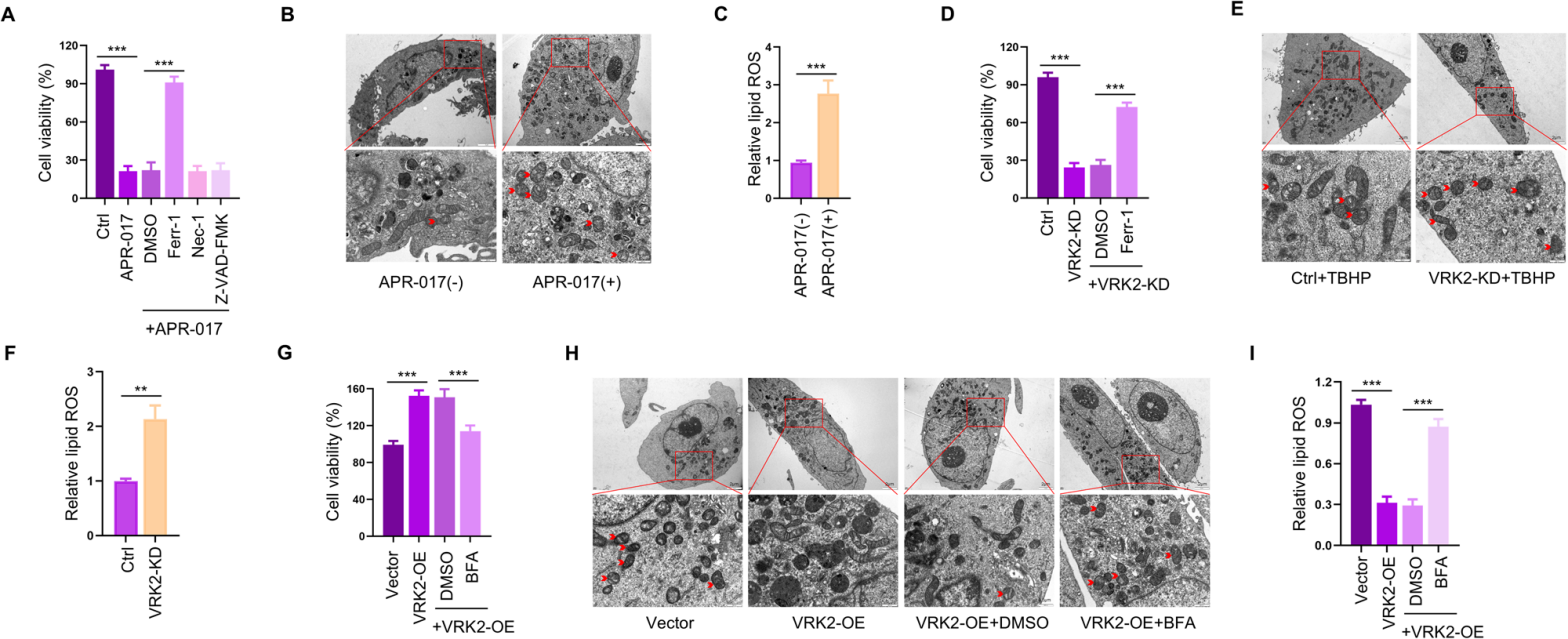
VRK2 protects PC cells from ferroptosis by promoting SLC7A11 trafficking. **A** PC cells exposed to APR-017 were treated with specific cell death inhibitors, ferrostatin-1 (8 μM), necrostatin-1 (6 μM), and Z-VAD-FMK (10 μM) for 24 h. The percentage of cell death was determined by the CCK8 assay. ***p < 0.001. **B** Representative TEM images of PC cells with APR-017 treatment. The red arrows are normal or morphologically abnormal mitochondria, manifesting as shrinkage of mitochondria, increased membrane density and reduced or vanished mitochondrial cristae. Low field ×2500, scale bar, 2 μm; high field ×12000, scale bar, 0.5 μm. **C** Relative lipid ROS levels were examined in VRK2-KO cells after treatment with APR-017. *n* = 3; ***p < 0.001. **D** Viability of shNC and shVRK2-transfected PC cells without or with ferrostatin-1 treatment was detected by CCK8 assay. *n* = 3; ***p < 0.001. **E** Representative TEM images of shNC or shVRK2-transfected PC cells with TBHP treatment(75 μM, 4 h). The red arrows are normal or morphologically abnormal mitochondria. Low field ×2500, scale bar, 2 μm; high field ×12000, scale bar, 0.5 μm. **F** Relative lipid ROS levels were examined in shNC or shVRK2-transfected PC cells after treatment with TBHP (75 μM, 4 h). *n* = 3; **p < 0.01. **G** Viability of control vector or exogenous VRK2-transfected PC cells treated without or with BFA. *n* = 3; ***p < 0.001. **H** Representative TEM images of control vector or exogenous VRK2-transfected PC cells treated without or with BFA. The red arrows are normal or morphologically abnormal mitochondria. Low field ×2500, scale bar, 2 μm; high field ×12000, scale bar, 0.5 μm. **I** Relative lipid ROS levels were examined in control vector or exogenous VRK2-transfected PC cells treated without or with BFA. *n* = 3; ***p < 0.001.

Since SLC7A11-mediated cystine uptake and subsequent biosynthesis of GSH detoxifies phospholipid peroxidation and protects cells from ferroptosis [26], we speculated that VRK2-deficient PC cells, vulnerable to GSH inhibition is associated with ferroptosis. To test the hypothesis, we applied ferroptosis inhibitor Ferrostatin-1 (Ferr-1), necrosis inhibitor Necrostatin-1 (Nec-1), or apoptosis inhibitor Z-VAD-FMK to treat VRK2-KO cells exposed to APR-017. The results of cck8 revealed that Ferr-1, rather than other cell death inhibitors, reversed the sensitization effect of APR-017 on VRK2-KO cells (Fig. 5A and Supplementary Fig. 5A). Transmission electron microscopy (TEM) confirmed that VRK2-KO cells treated with APR-017 exhibited increased shrunken mitochondria (Fig. 5B and Supplementary Fig. 5B). Lipid ROS, a classic biomarker of ferroptosis, was accumulated in VRK2-KO cells following APR-017 treatment (Fig. 5C and Supplementary Fig. 5C). These data suggested that VRK2-deficient PC cells underwent ferroptosis following GSH inhibition.

We have shown the difference between VRK2-WT cells and VRK2-KO cells in the sensitivity to GSH inhibition and noted that sensitive cells exhibited surged ROS levels following GSH inhibition (Fig. 1C, F). Consequently, we asked whether VRK2 was able to protect PC cells from ferroptosis induced by elevated ROS levels. We thus employed TBHP to supply ROS in VRK2-downregulating or VRK2-upregulating cells. When challenged with oxidative stress, VRK2 knockdown led to more cell death compared to the negative control group, and the increase in cell death could be mitigated by Ferr-1 (Fig. 5D and Supplementary Fig. 5D). Consistently, increased shrunken mitochondria and lipid ROS were observed in VRK2 knockdown cells in the presence of TBHP (Fig. 5E, F and Supplementary Fig. 5E, F). To add to this, VRK2 overexpression significantly attenuated TBHP-mediated cell death, which could be efficiently reversed by BFA treatment (Fig. 5G and Supplementary Fig. 5G). VRK2 overexpression could suppress the increase of shrunken mitochondria and lipid ROS induced by TBHP, while BFA treatment abolished VRK2-mediated alterations (Fig. 5H, I and Supplementary Fig. 5H, I), implying that VRK2 conferred the resistance of ferroptosis induced by ROS through facilitating the trafficking of SLC7A11.

### VRK2 expression stratifies the response of PC to GSH-targeted treatment

**Fig. 6:**
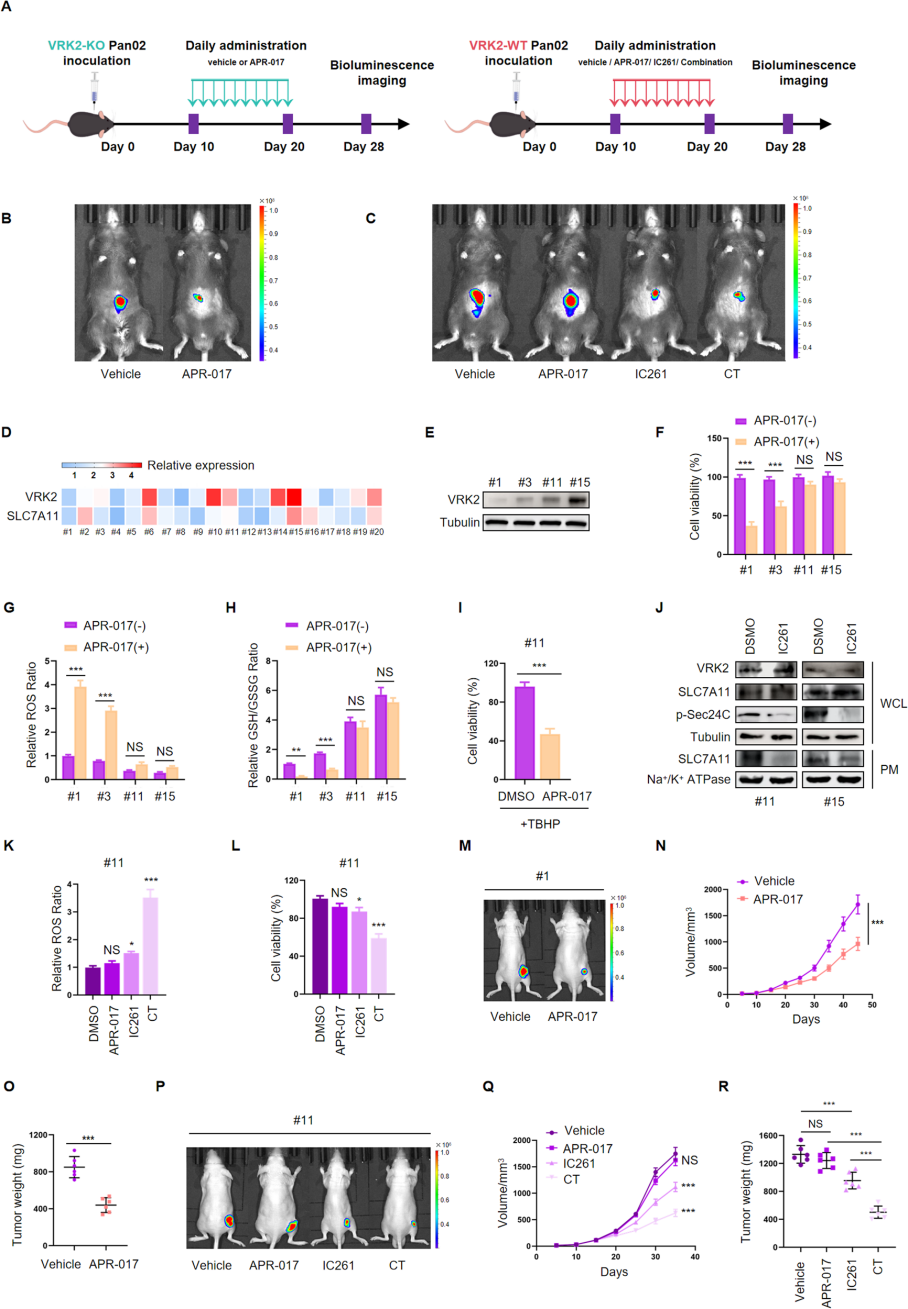
VRK2 expression stratifies the response of PC to GSH-targeted treatment. **A** Schematic illustration of the therapy schedule for APR-017, IC261, or combination therapy in the orthotopic pancreatic cancer mouse model using the Pan02 cell line. **B** VRK2-deficient orthotopic pancreatic cancer models were intraperitoneally injected with APR-017 (100 mg/kg) and then assessed by IVIS imaging system. **C** VRK2-expressing orthotopic pancreatic cancer models were intraperitoneally injected with APR-017 (100 mg/kg) and IC261 (0.6 mg/kg) alone or in combination and then assessed by IVIS imaging system. **D** Heatmap showing relative expression of VRK2 to Tubulin in whole-cell extracts of PC PDCs. **E** Immunoblotting for VRK2 in whole-cell extracts of PC PDCs. **F**–**H** Viability (**F**), relative ROS levels (**G**), and relative GSH levels (**H**) of PC PDCs were examined after exposure to 20 μM APR-017 for 48, 48, 24 h, respectively. *n* = 3; NS no significant. **p < 0.01. ***p < 0.001. **I** PC PDCs with high expression of VRK2 were exposed to TBHP (100 μM, 4 h) or co-treated with APR-017 (50 μM, 24 h). Viability of PDCs was evaluated at 24 h by CCK8 assay. *n* = 3; ***p < 0.001. **J** The expression of SLC7A11 and p-Sec24C at the whole cell level and SLC7A11 at the plasma membrane level in PC PDCs treated without or with IC261 (10 μM, 48 h) was examined by western blot. **K**, **L** Relative ROS levels (**K**) and viability (**L**) of PC PDCs were examined at 48 h after treatment with APR-017 (20 μM, 48 h) and IC261 (10 μM, 48 h) alone or in combination. *n* = 3; NS no significant. *p < 0.05. ***p < 0.001. **M** Nude mice with subcutaneously implanted xenografts derived from PDCs were intraperitoneally injected with APR-017 (100 mg/kg) and then assessed by IVIS imaging system. **N** Volume of tumors in the DMSO and APR-017 groups. Tumor volumes are presented as the mean ± SD, *n* = 6; ***p < 0.001. **O** The weight of the tumor in the DMSO and APR-017 groups. *n* = 6; ***p < 0.001. **P** Nude mice with subcutaneously implanted xenografts derived from PDCs were intraperitoneally injected with APR-017 (100 mg/kg) and IC261 (0.6 mg/kg) alone or in combination. Subcutaneous xenografts were assessed by the IVIS imaging system. **Q** Volume of tumors in APR-017 and IC261 alone or in combination groups. Tumor volumes are presented as the mean ± SD, *n* = 6; ns no significant. ***p < 0.001. **R** Weight of tumor in APR-017 and IC261 alone or in combination. *n* = 6; ns no significant. ***p < 0.001.

To further investigate the differential sensitivity to GSH inhibitors between VRK2-expressing and VRK2-deficient pancreatic tumors in vivo, we developed orthotopic pancreatic cancer models using Pan02 cells (Fig. 6A). Consistent with our hypothesis, APR-017 treatment significantly reduced tumor burden in VRK2-knockout models (Fig. 6B). Intriguingly, while VRK2 wild-type tumors were resistant to APR-017 monotherapy, combinatorial treatment with the VRK2 inhibitor IC261 restored their sensitivity to APR-017 (Fig. 6C). To validate the clinical relevance of these findings, we examined the expression of VRK2 and SLC7A11 in PC patient-derived cancer cells (PDCs). VRK2 expression at whole cell level was positively correlated with SLC7A11 expression at cell PM level (Fig. 6D). PDCs from four PC patients, of which two cases with VRK2 low expression and two cases with high expression, were utilized to perform the following functional experiments (Fig. 6E). Consistent with the results acquired from PC cell lines, PDCs with low expression of VRK2 were more sensitive to APR-017 treatment than PDCs with high expression of VRK2 (Fig. 6F). Compared to PDCs with high expression of VRK2, PDCs with low expression of VRK2 displayed a higher basal level of ROS and a lower basal level of GSH. APR-017 treatment led to increased ROS level and decreased GSH level in PDCs with low expression of VRK2 rather than PDCs with high expression of VRK2 (Fig. 6G, H). Interestingly, adding exogenous ROS induced by TBHP led to PDCs with high expression of VRK2 susceptibility to APR-017 treatment (Fig. 6I and Supplementary Fig. 6A), suggesting that PC cells with high expression of VRK2 have abundant antioxidant scavenging capacity to resist ROS accumulation induced by GSH inhibition. We then applied IC261 to test whether inhibition of VRK2 could make PDCs with high expression of VRK2 resensitization to GSH-targeting therapy. As shown in Fig. 6J, IC261 treatment led to decreased p-Sec24C expression and attenuated expression of SLC7A11 at the PM level in PDCs with high expression of VRK2. Notably, treatment with APR-017 alone affected neither ROS level nor cell viability, while treatment with IC261 alone slightly elevated ROS level and reduced cell viability. Combined treatment with IC261 and APR-017 resulted in a remarkable reduction in cell viability and a significant increase in ROS level when compared to the control or single treatment groups (Fig. 6K, L and Supplementary Fig. 6B, C).

To better simulate the clinical situation, a xenograft mouse model derived from PDCs with high and low expression of VRK2 was employed for further study. In xenografts derived from PDCs with low expression of VRK2, APR-017 treatment induced a prominent reduction in the volume and weight of the tumor (Fig. 6M–O and Supplementary Fig. 6D–F). In xenografts derived from PDCs with high expression of VRK2, the growth of the tumor was markedly suppressed in the group that received combined treatment with APR-017 and IC261, followed by the group that received IC261 treatment, and barely altered in the APR-017 group (Fig. 6P–R and Supplementary Fig. 6G–I). Overall, we conclude that PC patients with low expression of VRK2 are sensitive to GSH-targeting therapy, while PC patients with high expression of VRK2 are re-sensitive to GSH-targeting therapy following VRK2 inhibition, indicating genetic aberrance of VRK2 expression stratifies the response of PC to metabolism-targeted therapies.

## Discussion

Metabolism reprogramming has gained much attention since cancer cells have the ability to rewire metabolism to promote cell survival in response to environmental stress [27, 28]. Our findings demonstrated that GSH inhibition leads to synthetic lethality in VRK2-deficient PC cells, which exhibits impaired expression of SLC7A11 at PM level. VRK2 facilitates ER-to-Golgi transport of SLC7A11 by promoting the phosphorylation of Sec24C and its interaction with SLC7A11, thereby enhancing GSH synthesis and rendering resistance to ferroptosis. Furthermore, we found that the response of PC to GSH-targeted treatment is largely determined by stratified expression of VRK2, which provides a theoretical basis for individualized therapy targeting metabolism.

In our study, the dependency on GSH in VRK2-KO cells was identified using a synthetic lethal pharmacological screening strategy. We found that VRK2-deficient PC cells were sensitive to GSH inhibition induced by APR-017 and LCS3. LCS3 was previously uncovered to inhibit lung adenocarcinoma growth by targeting both GSR and TXNRD1 [19]. However, we suggested that the lethality induced by LCS3 in VRK2-KO PC cells is primarily dependent on inhibiting the activity of GSR rather than TXNRD1, for the following reasons. First, LCS3 treatment induced GSH inhibition in VRK2-KO cells, reflected by decreased levels of GSH/GSSG, whereas no significant effect on TXNRD1 activity was verified by the same concentration of LCS3. Second, given that the activation of GSR and TXNRD1 are involved in ROS elimination, if the LCS3-triggered lethality of VRK2-KO PC cells could be attributed to inhibiting the activity of both GSR and TXNRD1, then the level of ROS generated by LCS3 should have been much higher than those produced by the treatment of the TXNRD1 inhibitor auranofin. In contrast, our evidence showed that LCS3 induced generation of ROS was not more than that produced by auranofin. Third, the APR-017 and LCS3 induced increase in ROS, decrease in GSH, and inhibition of cell growth in VRK2-KO cells could be reversed by supplying a GSH compensator. Together, these findings suggest that VRK2-deficient PC cells exhibit a metabolic preference for GSH dependency, implying that VRK2 has the potential to regulate GSH biosynthesis.

It is reported that the GSH system plays an important role in maintaining redox homeostasis and thus supports cancer cells' survival in response to oxidative stress [29]. In addition to the GCL-mediated main biosynthetic pathway, GSH is also derived from compensatory mechanisms mainly mediated by GSR [5]. Our findings revealed that disrupting either of these pathways alone does not have a significant impact on cell viability. Nevertheless, their combined inhibition leads to synthetic lethality. This context-dependent sensitivity to GSH inhibition indicates that the viability of PC cells largely lies in the balance between ROS generation and endogenous antioxidant scavenging capacity. Based on results reported here, PC cells with VRK2 high expression can tolerate GSH inhibition because of its sufficient antioxidant capability which reflected by low basal level of ROS and high basal level of GSH. The addition of exogenous ROS results in resensitization of cells with high VRK2 expression to GSH inhibition, which is consistent with the fact that PC cells with low expression of VRK2 are susceptible to GSH inhibition attributes to its high basal ROS level, suggesting that cells in a state of oxidative stress have enhanced dependence on the GSH system. Thus, we have demonstrated that GSH-targeted therapy selectively kills PC cells in an oxidative stress-dependent manner and that the differences in basal ROS level and antioxidant scavenging capacity induced by stratified expression of VRK2 can well account for the heterogeneous sensitivity to GSH inhibition.

Finally, we investigated the molecular mechanism by which VRK2 promotes GSH biosynthesis and found that VRK2 drives the trafficking of SLC7A11 through phosphorylating Sec24C. In recent years, SLC7A11 has attracted increasing attention due to its critical role in the synthesis of GSH, which functions to prevent lipid peroxidation and protect cells from ferroptosis [30]. Dysfunction of SLC7A11 has been reported in diverse types of cancer, including PC, and is closely related to the malignant behavior of cancer [31, 32]. Studies have shown that the expression of SLC7A11 is regulated by multiple mechanisms, including transcription, epigenetics, and post-translational modification [33–35]. For example, the transcription factor Nrf2 binds to the antioxidant response element in the promoter region of SLC7A11 and then upregulates its expression [33]. Histone demethylase KDM3B enhances SLC7A11 expression by inhibiting H3K9 methylation [34]. Deubiquitylase OTUB1 reduces the ubiquitination and stabilizes the expression of SLC7A11 [35]. While the function of transmembrane protein SLC7A11 is primarily affected by its membrane localization, the mechanism regulating the intracellular transport and membrane location of SLC7A11 is rarely documented. Our results indicate that VRK2 phosphorylates COPII component Sec24C and promotes its interaction with SLC7A11, thus facilitating ER-to-Golgi transport and membrane expression of SLC7A11. We also observed that PC cells with upregulating expression of SLC7A11 at the PM level induced by VRK2 display decreased incidence of ferroptosis, which accounts for why PC cells with VRK2 high expression are insensitive to GSH inhibition alone. In addition, VRK2 overexpression confers PC cells resistant to ferroptosis induced by exogenous ROS through promoting ER-to-Golgi trafficking of SLC7A11, which echoes the findings that PC cells with high expression of VRK2 have an elevated basal level of GSH. These discoveries gain additional significance in light of recent evidence linking ferroptosis induction to enhanced immunotherapy efficacy, where GSH depletion-triggered ferroptosis can boost anti-tumor immunity through promoting the maturation of dendritic cells and the priming of CD8 + T cells [36]. Our identification of VRK2-mediated SLC7A11 trafficking suggests a potential immune evasion mechanism, as SLC7A11-high tumors often display T cell exclusion phenotypes [37], warranting future investigation into whether VRK2 expression could predict therapeutic response to combined ferroptosis inducers and immune checkpoint inhibitors.

Previous studies have shown that the binding to Sec24 is an important step for cargoes to undergo ER-to-Golgi trafficking [23]. Various mammalian isoforms of Sec24 contribute to the diversity of cargo selection in the process of vesicular transport. For instance, Sec24B has the ability to transport Vangl2 while Sec24C and D were responsible for the trafficking of membrin and syntaxin-5 [38, 39]. Of note, a recent investigation showed that Sec24C interacts with SLC6A14, a member of the solute carrier family, and promotes its vesicular trafficking to the PM [40]. Our data demonstrate that solute carrier SLC7A11 is also recognized by Sec24C for ER-to-Golgi transport and membrane location. Interestingly, the interaction of Sec24C and SLC7A11 is enhanced by VRK2-mediated phosphorylation, which is consistent with previous studies showing that the phosphorylation of Sec24C determines its affinity to cargo proteins and intracellular transport of cargoes [25]. Collectively, these findings suggest that VRK2 promotes phosphorylation of Sec24C and its interaction with SLC7A11, thereby driving the ER-to-Golgi trafficking of SLC7A11. Nevertheless, further investigations are warranted to elucidate the specific phosphorylating sites within Sec24C modified by VRK2.

In summary, our study establishes that VRK2 expression dictates PC cell vulnerability to GSH-targeted therapies through distinct mechanisms. In VRK2-high tumors, VRK2 promotes SLC7A11 trafficking from the ER to the Golgi, enhancing GSH synthesis and conferring resistance to GSH inhibition. This oncogenic role validates VRK2 as a therapeutic target in VRK2-overexpressing PC, where pharmacological inhibition restores sensitivity. Conversely, VRK2-low PC cells exhibit impaired SLC7A11 membrane localization and intrinsic oxidative stress, creating a therapeutic window for GSH inhibition. Consequently, VRK2 expression emerges as a dual-function biomarker: its targeted inhibition shows therapeutic efficacy in VRK2-high malignancies, while its deficiency serves as a predictive indicator for metabolic-targeted interventions. These opposing yet complementary mechanisms highlight the critical importance of VRK2 expression profiling for implementing precision oncology approaches in PC management.

## Supplementary information

Supplementary Figure

Supplementary Materials and Methods

Table S1

Raw Data

## Data Availability

All datasets generated and analyzed during this study are included in this published article and its supplementary information files. Any additional data are available from the corresponding author upon reasonable request.
