# Supplementary material for: Vaccinia-related kinase 2 inhibition elicits vulnerability of glutathione metabolism in pancreatic cancer
Source: Cell Death Dis. 2026 Mar 19;17(1):325. doi: 10.1038/s41419-026-08573-9 (PMC13039163; doi:10.1038/s41419-026-08573-9)
Supplement: Supplementary file 1 — Supplementary Figure [file 41419_2026_8573_MOESM1_ESM.docx]

**Supplementary Figure Legends**

**
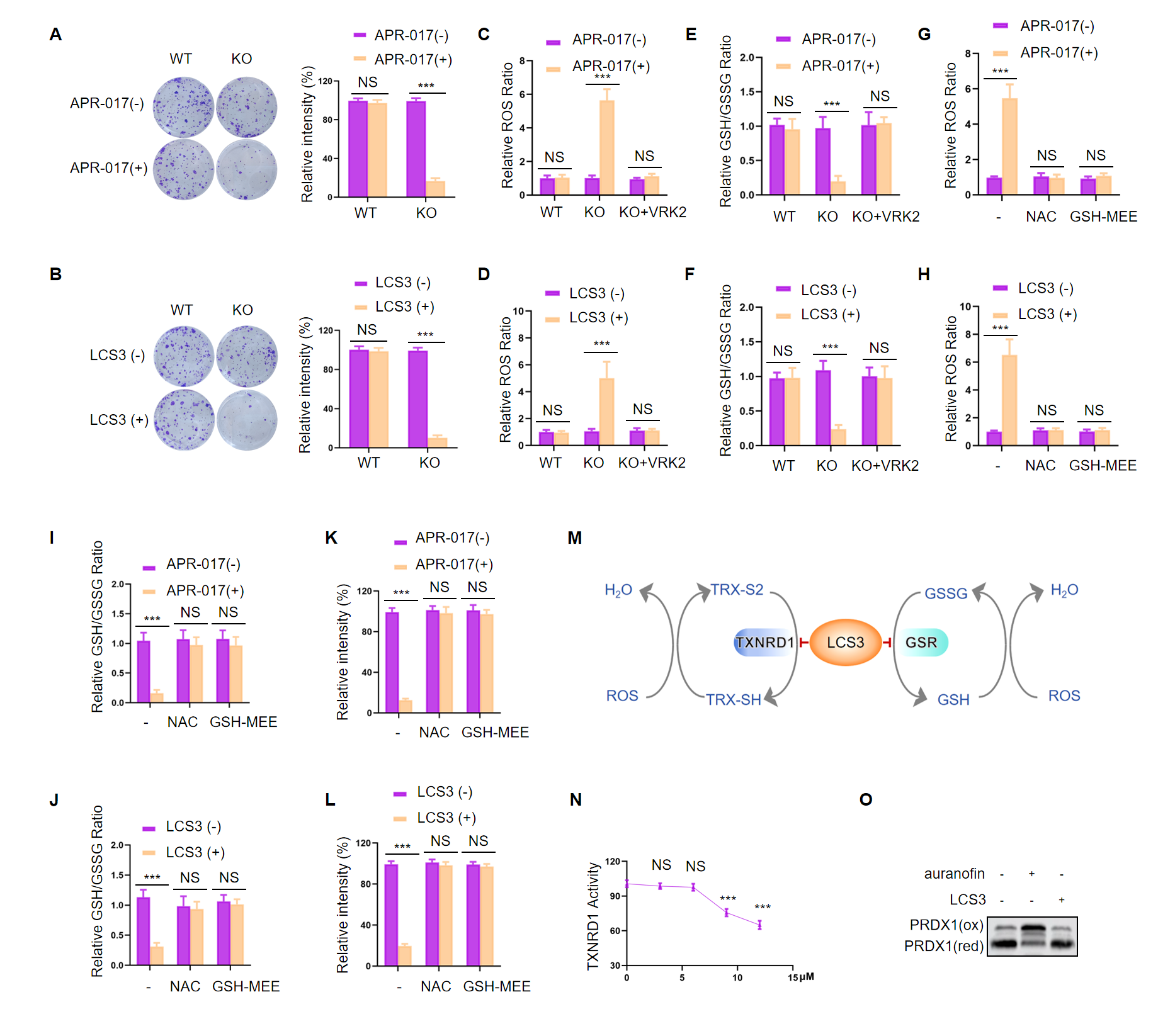
**

**Supplementary Fig.1 VRK2-deficient PC cells are sensitive to GSR inhibitor**

**A, B** VRK2-WT and VRK2-KO PC cells were exposed to APR-017 (15 μM, 72h) or LCS3 (10 μM, 72h) and then cultured for 14 days. Cell growth was assessed by colony formation assay. n=3; NS, no significant. ***p <0.001. **C-F** Relative ROS levels (C, D) and relative GSH levels (E, F) were examined in VRK2-WT, VRK2-KO and VRK2 restoring VRK2-KO cells after treatment with 40 μM APR-017 or 15 μM LCS3 for 48, 48, 24, 24h, respectively. n=3; NS, no significant. ***p <0.001. **G-L** Relative ROS levels (J, K), relative GSH levels (L, M) and cell viability (N, O) were tested in VRK2-KO cells after treatment with APR-017 (40 μM) or LCS3 (15 μM) for 48, 48, 24, 24, 72, 72h, respectively, without or with co-treatment of NAC (10 mM) or GSH-MEE (5 mM). n=3; NS, no significant. ***p <0.001. **M** Schematic showing dual inhibition of LCS3 on the activity of GSR and TXNRD1. **N** Relative TrxR activities were detected in PC cells with different concentration of LCS3 treatment for 24h. n=3; NS, no significant. ***p <0.001. **O** Redox western blot showing PRDX1 redox status in VRK2-KO cells treated with auranofin (6 μM) or LCS3 (10 μM).

**
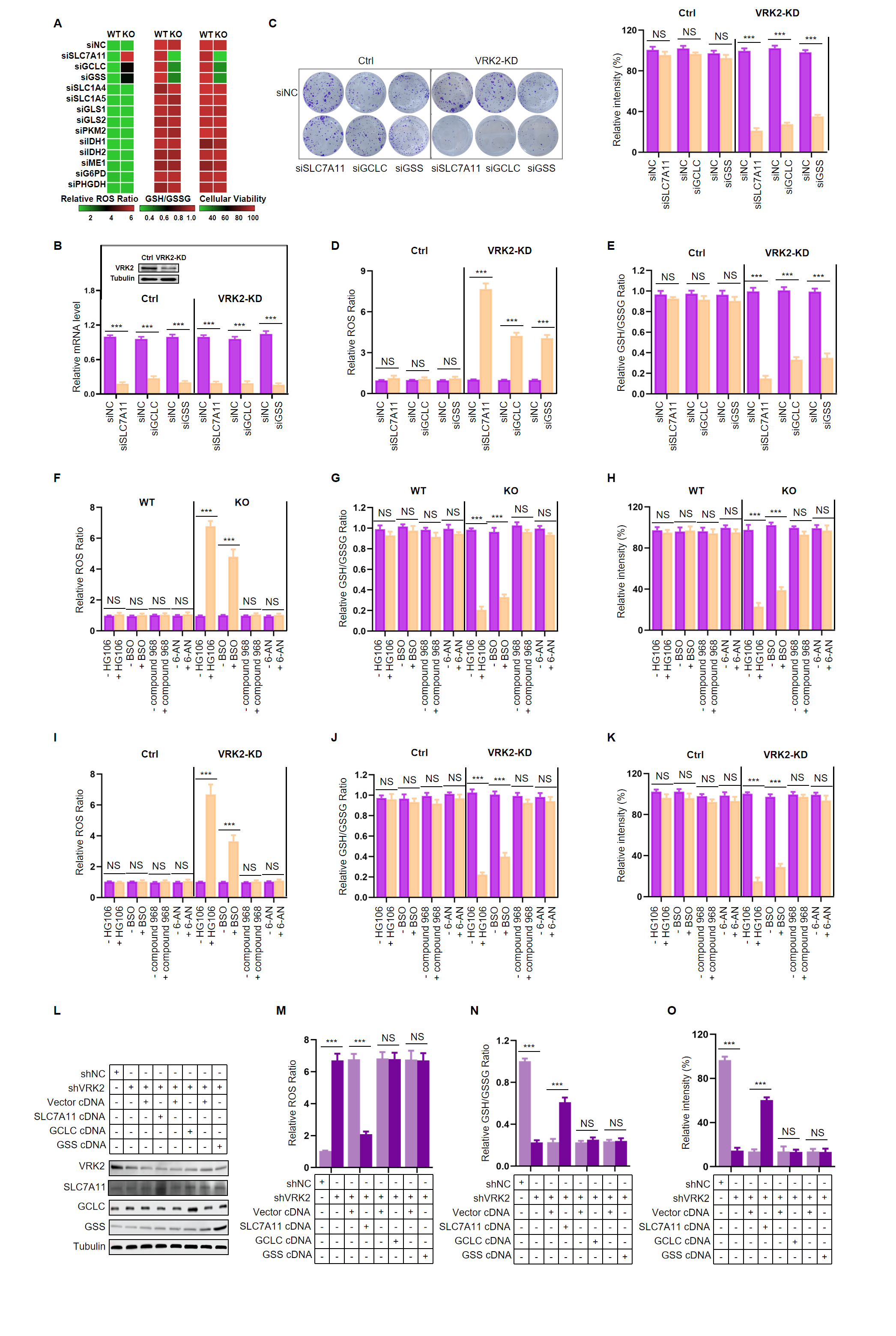
**

**Supplementary Fig.2 SLC7A11 is a key target involved in VRK2 rewiring GSH metabolism**

**A** Heatmap showing relative ROS levels, GSH levels and cell viability in VRK2-WT and VRK2 KO cells at 48, 24, and 48 h, after knockdown of GSH pathway genes. **B** VRK2 knockdown in PC cells was validated by western blot (upper panel), and the downregulation of SLC7A11, GCLC and GSS were confirmed by RT-PCR (lower panel). n=3; ***p <0.001. **C** Viability of shNC and shVRK2 transfected PC cells with SLC7A11, GCLC, GSS knockdown, respectively. n=3; NS, no significant. ***p <0.001. **D, E** Relative ROS levels (D) and relative GSH levels (E) were examined in shNC and shVRK2 transfected PC cells with SLC7A11, GCLC, GSS knockdown, respectively. n=3; NS, no significant. ***p <0.001. **F-H** Relative ROS levels (F), relative GSH levels (G) and viability (H) were assessed in VRK2-WT and VRK2 KO cells after treatment of HG106 (2 μM), BSO (20 μM), compound 968 (10 μM) and 6-AN (10 μM) for 48, 24, 48h, respectively. n=3; NS, no significant. ***p <0.001. **I-K** Relative ROS levels (I), relative GSH levels (J) and viability (K) were assessed in shNC and shVRK2 transfected PC cells after treatment of HG106 (4 μM), BSO (30 μM), compound 968 (20 μM) and 6-AN (15 μM) for 48, 24, 48h, respectively. n=3; NS, no significant. ***p <0.001. **L** SLC7A11, GCLC and GSS overexpression was validated by western blot in shNC and shVRK2 transfected PC cells. **M-O** Relative ROS levels (M), relative GSH levels (N) and viability (O) were evaluated in shNC and shVRK2 transfected PC cells at 48, 24, 48h, after upregulating the expression of SLC7A11, GCLC or GSS, respectively. n=3; NS, no significant. ***p <0.001.


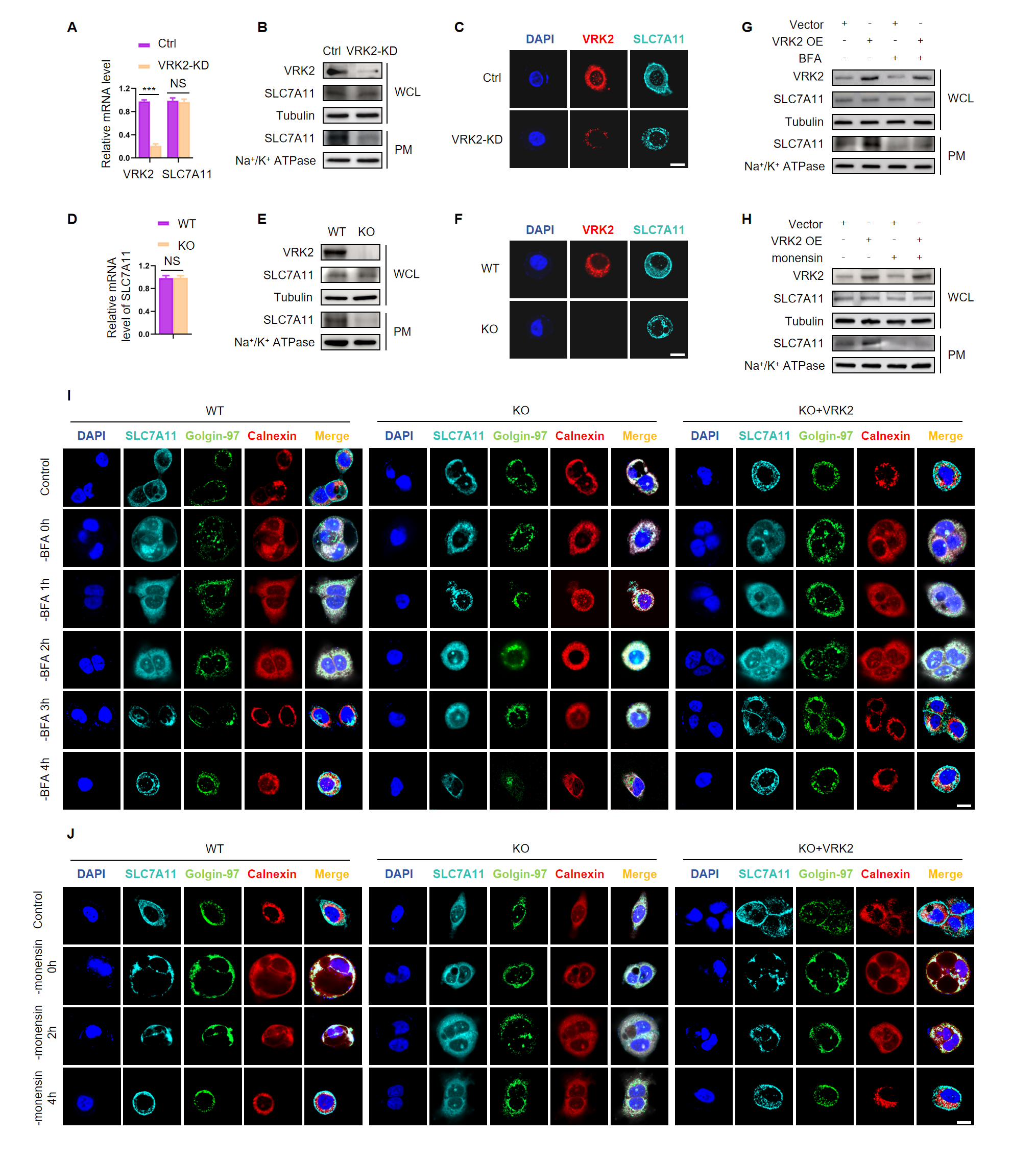


**Supplementary Fig.3 VRK2 promotes ER-to-Golgi trafficking of SLC7A11 and its expression on cell membrane**

**A** mRNA of VRK2 and SLC7A11 in shNC or shVRK2 transfected PC cells tested by RT-qPCR. n = 3; NS, no significant. ***p <0.001. **B** Immunoblot for protein expression of SLC7A11 at whole cell level and plasma membrane level in PC cells expressing shNC or shVRK2; WCL, whole cell lysate; PM, plasma membrane. **C** Subcellular location of SLC7A11 in PC cells expressing shNC or shVRK2 was detected by immunofluorescence; Scale bar indicates 20 μm. **D** mRNA of SLC7A11 in VRK2-WT and VRK2-KO PC cells. n = 3; NS, no significant. **E** Immunoblot for protein expression of SLC7A11 at whole cell level and plasma membrane level in VRK2-WT and VRK2-KO PC cells; WCL, whole cell lysate; PM, plasma membrane. **F** Subcellular location of SLC7A11 in VRK2-WT and VRK2-KO PC cells was detected by immunofluorescence; Scale bar indicates 20 μm. **G, H** Immunoblot for protein expression of SLC7A11 at whole cell level and plasma membrane level in control vector or exogenous VRK2 transfected PC cells treated without or with BFA (5 μg/ml, 1h) or monensin (5 μM, 6h). **I** ER-to-Golgi trafficking of SLC7A11 in VRK2-WT, VRK2-KO and VRK2 restoring VRK2-KO cells. Cells were treated with 5 μg/ml BFA for 1 hour and then analyzed by laser scanning confocal microscope at different times after BFA removal. Endoplasmic reticulum was labeled by calnexin antibody. Golgi apparatus was labeled by Golgin-97 antibody. Scale bar indicates 20 μm. **J** Golgi-to-PM transport of SLC7A11 in VRK2-WT, VRK2-KO and VRK2 restoring VRK2-KO cells. Cells were treated with 5 μM monensin for 6 hour and then analyzed by laser scanning confocal microscope at different times after monensin removal. Scale bar indicates 20 μm.


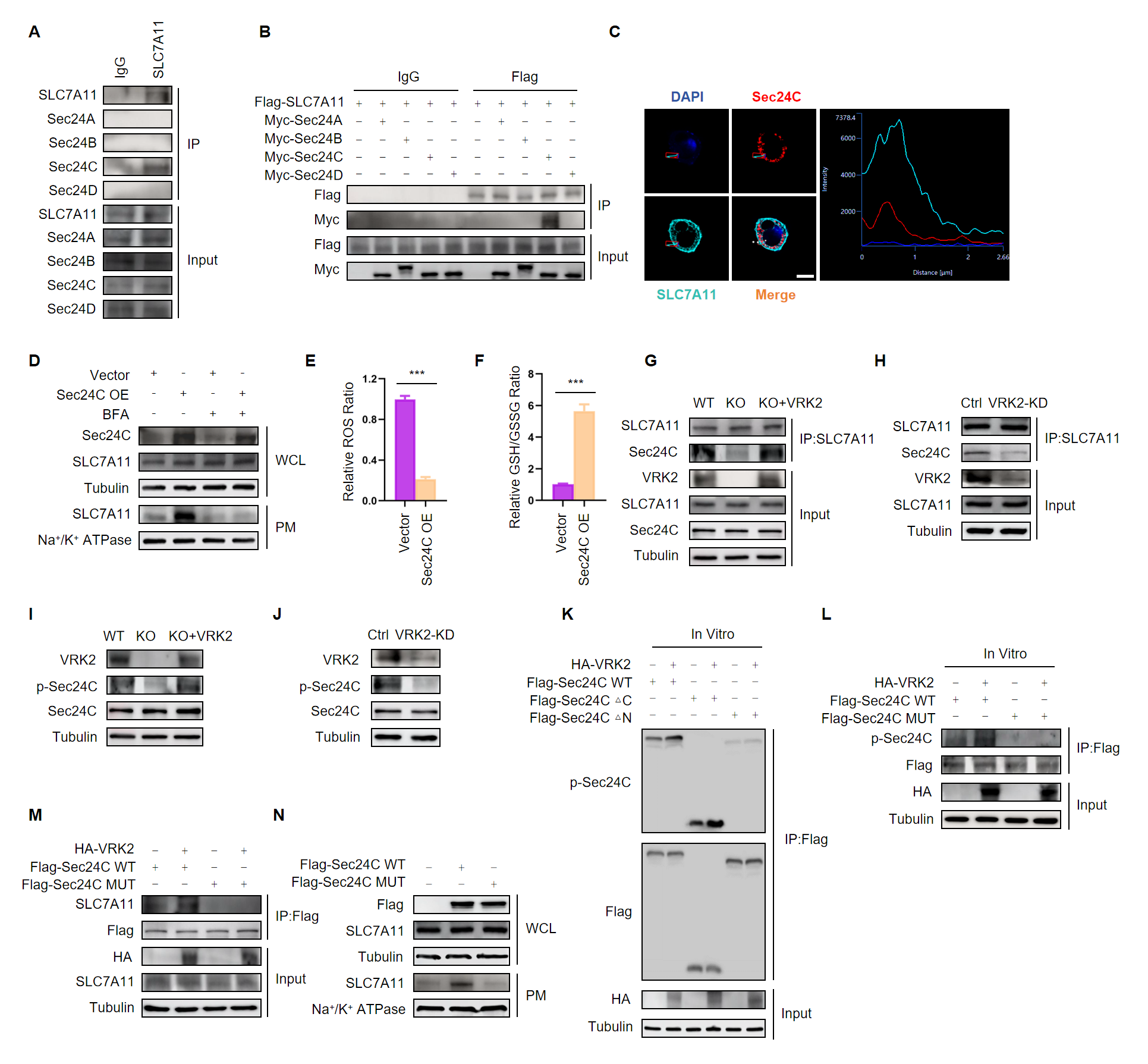


**Supplementary Fig.4** **VRK2 promotes the transport of SLC7A11 by phosphorylation of Sec24C**

**A** The interaction of endogenous SLC7A11 and Sec24 isoforms were detected by IP. **B** PC cells were transfected with exogenous SLC7A11 and Sec24 isoforms. The interaction of exogenous SLC7A11 and Sec24 isoforms were detected by IP. **C** The co-localization of SLC7A11 and Sec24C was examined by immunofluorescence (left panel). Dimensions result of confocal showed that the intensity of turquoise and red has the same variation tendency(right panel). Scale bar indicates 20 μm. **D** Immunoblot for protein expression of SLC7A11 at whole cell level and plasma membrane level in control vector or exogenous Sec24C transfected PC cells treated without or with BFA (5 μg/ml, 1h) or monensin (5 μM, 6h); WCL, whole cell lysate; PM, plasma membrane. **E, F** Relative ROS levels (E) and relative GSH levels (F) were evaluated in PC cells transfected with control vector or exogenous Sec24C. n=3. ***p <0.001. **G** The interaction of SLC7A11 and Sec24 isoforms in VRK2-WT, VRK2-KO and VRK2 restoring VRK2-KO cells. **H** The interaction of SLC7A11 and Sec24 isoforms in PC cells expressing shNC or shVRK2. **I** Immunoblot for protein expression of p-Sec24C and total-Sec24C in VRK2-WT, VRK2-KO and VRK2 restoring VRK2-KO cells. **J** Immunoblot for protein expression of p-Sec24C and total-Sec24C in PC cells expressing shNC or shVRK2. **K** VRK2 phosphorylates Sec24C *in vitro*. Flag-Sec24C WT, Flag-Sec24C△C or Flag-Sec24C△N proteins were incubated *in vitro* with immunoprecipitates isolated from PC cells transfected with constructs encoding HA-VRK2 and then analyzed by western blot using indicated antibodies. **L** Flag-Sec24C WT or Flag-Sec24C MUT protein was incubated *in vitro* with immunoprecipitates isolated from PC cells transfected with constructs encoding HA-VRK2 and then analyzed by western blot using indicated antibodies. **M** PC cells were transfected with HA-VRK2 and Flag-Sec24C WT or Flag-Sec24C MUT. The interaction of SLC7A11 and Flag-Sec24C WT or Flag-Sec24C MUT was analyzed by IP. **N** PC cells were transfected with Flag-Sec24C WT or Flag-Sec24C MUT. The expression of SLC7A11 at whole cell level and plasma membrane level was then tested by western blot; WCL, whole cell lysate; PM, plasma membrane.


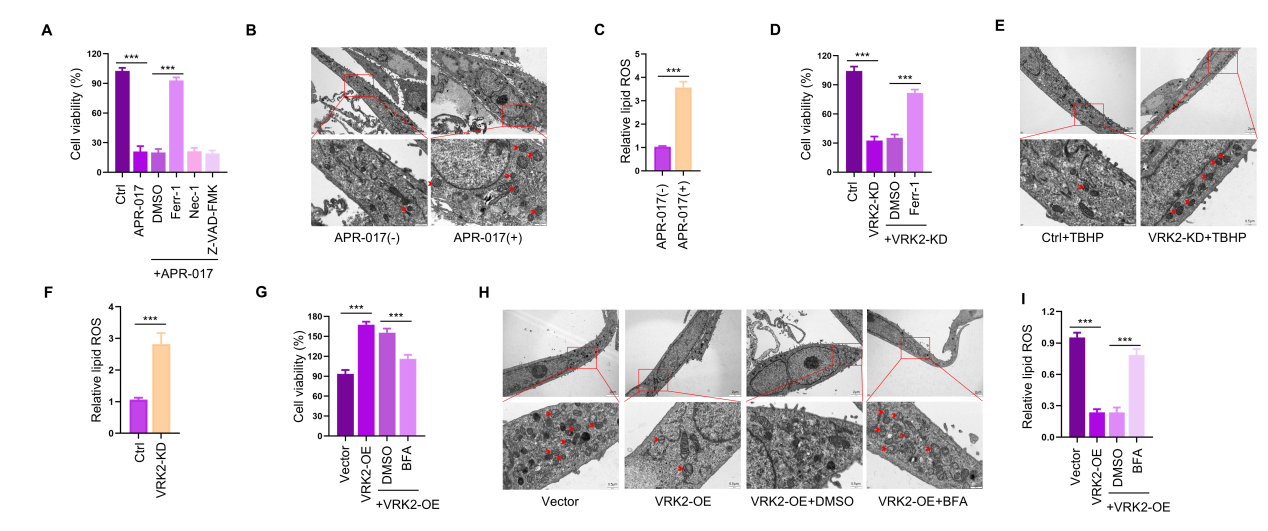


**Supplementary Fig.5 VRK2 protects PC cells from ferroptosis by promoting SLC7A11 trafficking**

**A** PC cells exposed to APR-017 were treated with specific cell death inhibitors, ferrostatin-1 (10 μM), necrostatin-1 (5 μM), Z-VAD-FMK (10 μM) for 24 h. The percentage of cell death was determined by CCK-8 assay. ***p <0.001. **B** Representative TEM images of PC cells with APR-017 treatment. The red arrows are normal or morphologically abnormal mitochondria, manifesting as shrinkage of mitochondria, increased membrane density and reduced or vanished mitochondrial cristae. Low field ×2500, scale bar, 2μm; high field ×12000, scale bar, 0.5μm. **C** Relative lipid ROS levels were examined in VRK2-KO cells after treatment with APR-017. n=3; ***p <0.001. **D** Viability of shNC and shVRK2 transfected PC cells without or with ferrostatin-1 treatment was detected by CCK-8 assay. n=3; ***p <0.001. **E** Representative TEM images of shNC or shVRK2 transfected PC cells with TBHP treatment(75 μM, 4h). The red arrows are normal or morphologically abnormal mitochondria. Low field ×2500, scale bar, 2μm; high field ×12000, scale bar, 0.5μm. **F** Relative lipid ROS levels were examined in shNC or shVRK2 transfected PC cells after treatment with TBHP (75 μM, 4h). n=3; **p <0.01. **G** Viability of control vector or exogenous VRK2 transfected PC cells treated without or with BFA. n=3; ***p <0.001. **H** Representative TEM images of control vector or exogenous VRK2 transfected PC cells treated without or with BFA. The red arrows are normal or morphologically abnormal mitochondria. Low field ×2500, scale bar, 2μm; high field ×12000, scale bar, 0.5μm. **I** Relative lipid ROS levels were examined in control vector or exogenous VRK2 transfected PC cells treated without or with BFA. n=3; ***p <0.001.


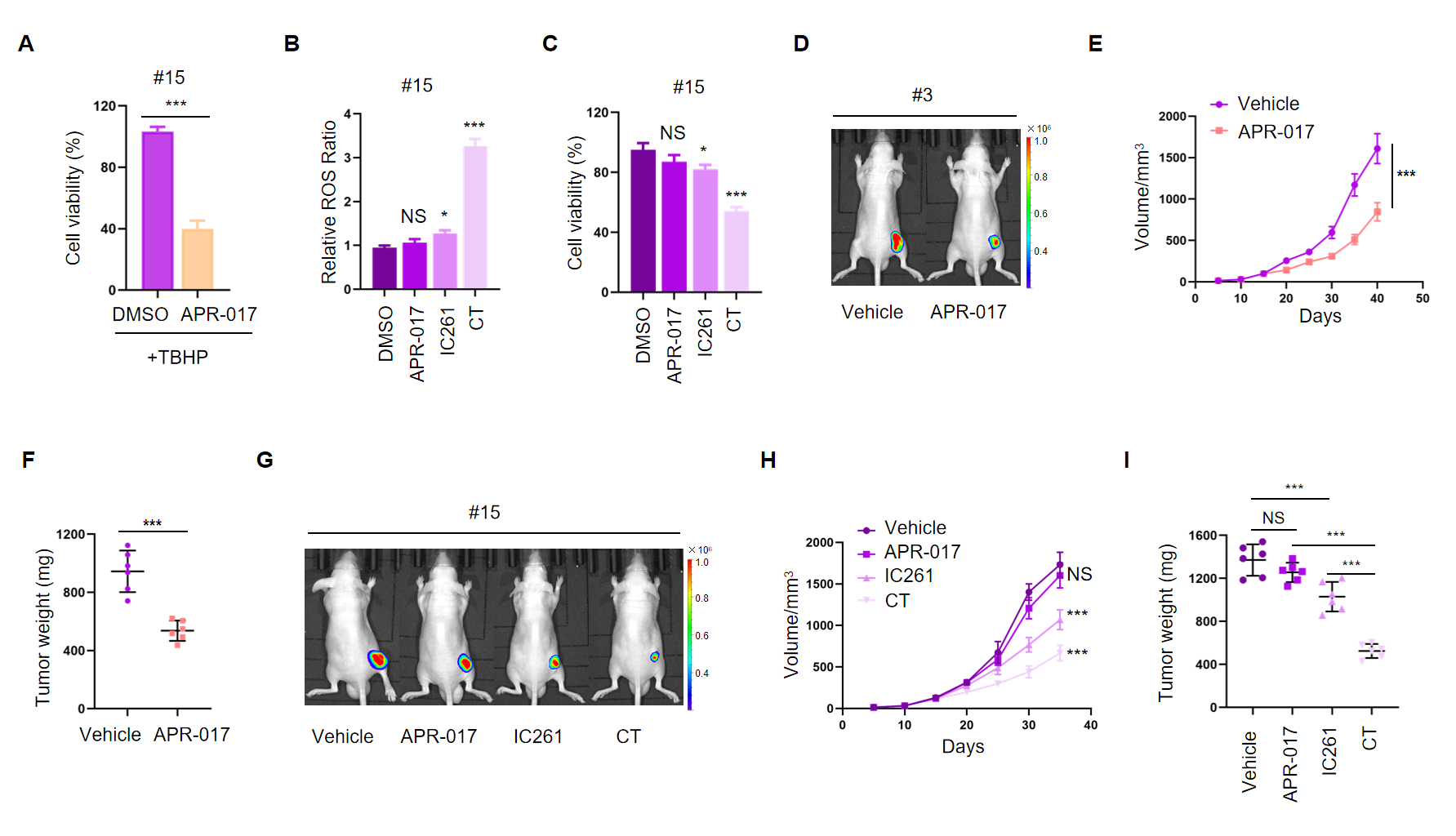


**Supplementary Fig.6 VRK2 expression stratifies the response of PC to GSH-targeted treatment**

**A** PC PDCs with high expression of VRK2 were exposed to TBHP (100 μM, 4h) or co-treated with APR-017 (70 μM, 24h). Viability of PDCs was evaluated at 24h by CCK-8 assay. n=3; ***p <0.001. **B, C** Relative ROS levels (B) and viability (C) of PC PDCs were examined at 48h after treatment with APR-017 (40 μM, 48h) and IC261 (25 μM, 48h) alone or in combination. n=3; NS, no significant. *p <0.05. ***p <0.001. **D** Nude mice with subcutaneously implanted xenografts derived from PDCs were intraperitoneally injected with APR-017 (100 mg/kg) and then assessed by IVIS imaging system. **E** Volume of tumors in the DMSO and APR-017 groups. Tumor volumes are presented as the mean ± SD, n=6; ***p < 0.001. **F** Weight of tumor in DMSO and APR-017 groups. n=6; ***p<0.001. **G** Nude mice with subcutaneously implanted xenografts derived from PDCs were intraperitoneally injected with APR-017 (100 mg/kg) and IC261 (0.6mg/kg) alone or in combination. Subcutaneous xenografts were assessed by IVIS imaging system. **H** Volume of tumors in APR-017 and IC261 alone or in combination groups. Tumor volumes are presented as the mean ± SD, n=6; ns, no significant. ***p < 0.001. **I** Weight of tumor in APR-017 and IC261 alone or in combination. n = 6; ns, no significant. ***p <0.001.
