# Supplementary material for: Vaccinia-related kinase 2 inhibition elicits vulnerability of glutathione metabolism in pancreatic cancer
Source: Cell Death Dis. 2026 Mar 19;17(1):325. doi: 10.1038/s41419-026-08573-9 (PMC13039163; doi:10.1038/s41419-026-08573-9)
Supplement: Supplementary file 2 — Supplementary Materials and Methods [file 41419_2026_8573_MOESM2_ESM.docx]

**Supplementary Materials and methods**

**Plasmids, antibodies and reagents**

VRK2 short hairpin RNA (shVRK2) plasmid, ligated in pGV248 vector, VRK2 overexpressed plasmid, SLC7A11 overexpressed plasmid, GCLC overexpressed plasmid, GSS overexpressed plasmid, full-length Sec24C overexpressed plasmid, truncated Sec24C overexpressed plasmid, ligated in pGV492 vector, were purchased from Shanghai GeneChem Technologies (Shanghai, China). The plasmid of Sec24C mutant was generated by site-directed mutagenesis. SLC7A11 small interfering RNA (siSLC7A11, #L-007612-01), GCLC small interfering RNA (siGCLC, L-009212-00), GSS small interfering RNA (siGSS, L-009586-00) were purchased from Dharmacon (Shanghai, China).

Anti-VRK2 (# ab58052), Anti-Tubulin (ab52866), Anti-Na^+^/K^+^ ATPase(#76020),

Anti-PRDX1 (#ab109506), Anti-SLC7A11 (#ab307601), Anti-GCLC(#ab207777), Anti-GSS(#ab266342), Anti-Sec24C(ab122633) were purchased from Abcam (Cambridge, UK). Anti-HA (#51064-2-AP), Anti-MYC (#16286-1-AP), Anti-Flag (#20543-1-AP), Anti-Sec24A(#15958-1-AP) were purchased from proteintech (Chicago, USA). Anti-Sec24B(#A304-876A), Anti-Sec24D(#13673-1-AP) was purchased from ThermoFisher (MA, USA). LCS3(#5181525) was purchased from Chembridge(CA, USA). APR-017(#S7723), HG106(#E1080), BFA(#S7046), monensin(S2324), Nec-1(S8037), Z-VAD-FMK(#S7023), IC261(#S8237) were purchased from Selleck (Shanghai, CHN). NAC (#A7250-25G), GSH-MEE (#353905), TBHP(#458139), BSO(#B2515), compound 968(#5.06073), 6-AN(#A68203), Auranofin(#A6733), Ferr-1(#SML0583) were purchased from sigma-aldrich (San Francisco, USA). DMSO (#BP231-100), Lipofectamine™ 3000 Transfection Reagent (#L3000015) was purchased from ThermoFisher (MA, USA). Protein A/G PLUS-agarose (#sc-2003) was purchased from Santa Cruz Biotechnology (Dallas, TX).

**CRISPR-Cas9 knockout screen**

For the deficiency of VRK2 in CFPAC-1 and SW1990 cells, the single guide RNAs (sgRNAs) were designed by applying the online CRISPR design tool (Red CottonTM, Guangzhou, China, https://en.rc-crispr.com/). To design the specific target sequences for sgRNA synthesis, the primers were: VRK2#1 forward (5' to 3') :TTGATTCCTACTACCATAAAAGG; VRK2#1 reverse (5' to 3') CCTTACCATCTTGATTAGAGAGG; VRK2#2 forward (5' to 3') AGTGTATGGGCTGTGATGTCAGG; VRK2#2 reverse (5' to 3') TGTATTGCCAGCTATTACCCTGG. For VRK2 knockout in Pan02 cells, the following primer sets were designed: VRK2#1 forward (5' to 3') : tgatatggaaggaaaccggtggg; VRK2#1 reverse (5' to 3') gcaagatgatcggctctggaggg; VRK2#2 forward (5' to 3') tccaaaagtggatacaacagagg; VRK2#2 reverse (5' to 3') ataccagattatattcaccaggg. The oligonucleotide pairs for both targeting sites were annealed and ligated into the YKO-RP003 vector (for CFPAC-1/SW1990) or CRISPR-V2 vector (for Pan02) (Ubigene Biosciences Co., Ltd., Guangzhou, China). CFPAC-1, SW1990, and Pan02 cells were transfected with sgRNA-containing plasmids using Lipofectamine 3000 reagent. After transfection for 48 hours, the cells were treated with puromycin in 1 mg/mL for 72 hours. The surviving cells were sorted into single monoclones and inoculated in 96-well plates. monoclonal screening was conducted after 2-4 weeks and selected VRK2-KO clones were validated by PCR and Sanger sequencing.

**Drug Library Screen**

VRK2-WT and VRK2-KO PC cells were seeded in 96-well plates and cultured for 24 hours. A panel of agents [including 281 metabolism compounds(Table S1)] at a concentration of 10 μM were added to treat these cells. After 3 days, cell viability was evaluated using the CellTiter-Glo Luminescent Cell Viability Assay (Promega). A multiscan spectrum (Bio-Rad) was used for the measurement of luminescence. The cell viability relative to that of cells exposed to solvent (DMSO) was calculated by the luminescence reading. If the viability of VRK2-KO cells is less than 40% while which of VRK2-WT cells is greater than 80%, candidate agents are screened out.

**Western blotting, qRT-PCR, Co-immunoprecipitation (Co-IP), cell colony formation assay, cell viability assay and immunofluorescent staining**

Western blotting, qRT-PCR, Co-immunoprecipitation (Co-IP), cell colony formation assay, cell viability assay and immunofluorescent staining were conducted as previously described[1]. The following primers were used for qRT-PCR: Human VRK2 (F:5’-GTGGATAGAACGCAAACAACTTG-3’; R: 5’-CGGATACCTAATTGCAGGACAGT-3’); Human SLC7A11 (F: 5’-TCAGAAGCTTATTTAATGGTGCG-3’; R: 5’-GTGGTTTTGGATTCAGTGAGAAG-3’); Human GCLC (F: 5’-TCCAGGTGACATTCCAAGCC-3’; R: 5’-GAAATCACTCCCCAGCGACA-3’); Human GSS (F: 5’-GATGGACTTCAACCTGCTAGTG-3’; R: 5’-GTCAAAGAGACGAGCGGTAAA-3’); Humanβ-actin (F: 5’- TGACGTGGACATCCGCAAAG-3’; R: 5’- CTGGAAGGTGGACAGCGAGG-3’).

**Redox western blotting**

For redox western blotting, PC cells with auranofin or LCS3 treatment were dissolved in alkylation buffer (40 mM HEPES, 50 mM NaCl, 1mM EGTA, HALT^TM^ protease and phosphatase inhibitors, pH7.4). The cells were then washed with cold PBS, followed by dissolutionvin alkylation buffer containing N-Ethylmaleimide for 10 minutes at room temperature. 10% CHAPS was added to the cells and incubated for 10 minutes. The cellular lysates were harvested and incubated on ice for 30 minutes. Samples were prepared with Laemmli sample buffer and separated by sodium dodecyl sulphate-polyacrylamide gel electrophoresis (SDS-PAGE). A Bio-Rad ChemiDoc MP imager was applied to analyze the intensity of each band.

***In Vitro* Kinase Assay**

The PC cells transfected with HA-VRK2 and Flag-Sec24C(full-length) or Flag-Sec24C(truncated) were harvested and lysed in the lysis buffer (150mM NaCl, 10mM Tris-Cl pH 7.4, 1% Nonidet P-40, 1mM EDTA, 1% SDS and 0.5% deoxycholic acid). The protein lysates were immunoprecipitated with anti-Flag antibody and protein G-Sepharose beads at 4 °C for 4 h. The beads were washed twice with lysis buffer followed by kinase buffer (50 mM Tris pH 7.5, 5mM MgCl2, 1 mM DTT). Phosphorylation reaction was then carried out in 20 μl of kinase buffer (20 mM Tris PH 7.4, 5 mM MgCl2, 0.5 mM dithiothreitol, 150 mM KCl, and γ-^32^P ATP) at 30 °C for 30 min. The reactions were dissolved in Laemmli sample Buffer and visualized with autoradiography.

**Detection of TrxR Activity**

Thioredoxin Reductase Assay Kit (Abcam) was used for the detection of TrxR activity. PC cells were seeded in 10 cm dishes at the specified density. Twenty-four hours later, the cells were treated with the indicated concentrations of agents. The processed cells were washed with cold PBS, lysed in buffer and then centrifugated with a centrifuge. The supernatant was added with a TrxR inhibitor and incubated for 30 min at room temperature. Absorbance was measured using a multiscan spectrum.

**Establishment of PC Cancer PDCs**

PC samples were acquired from 20 PC patients who underwent surgery at the second affiliated hospital of zhejiang university school of medicine and were cultured in vitro. Informed consent was obtained from the patients and the study protocol was approved by the Ethics Committee of the Second Affiliated Hospital, Zhejiang University School of Medicine. To establish PC PDCs, the PC samples were cut into small pieces, trypsinized, and then pelleted by centrifugation followed by incubation in hemolysis buffer (0.75% NH4Cl and 17 mM Tris-HCl, pH 7.65). The pellets were then washed with PBS and cultured in RPMI 1640. Thereafter, the culture medium was discarded and replaced by DMEM to remove lymphocytes. Adherent cells were cultured in RPMI 1640 containing 10% FBS until multiple colonies formation. During culture, the cells were treated with 0.05% trypsin-EDTA to remove fibroblasts and other types of cells.

**References**

1. Zhu H, Li Q, Zhao Y, Peng H, Guo L, Zhu J, et al. Vaccinia-related kinase 2 drives pancreatic cancer progression by protecting Plk1 from Chfr-mediated degradation. Oncogene. 2021;40(28):4663-74.
