# Supplementary material for: Vaccinia-related kinase 2 inhibition elicits vulnerability of glutathione metabolism in pancreatic cancer
Source: Cell Death Dis. 2026 Mar 19;17(1):325. doi: 10.1038/s41419-026-08573-9 (PMC13039163; doi:10.1038/s41419-026-08573-9)

Fig1

A

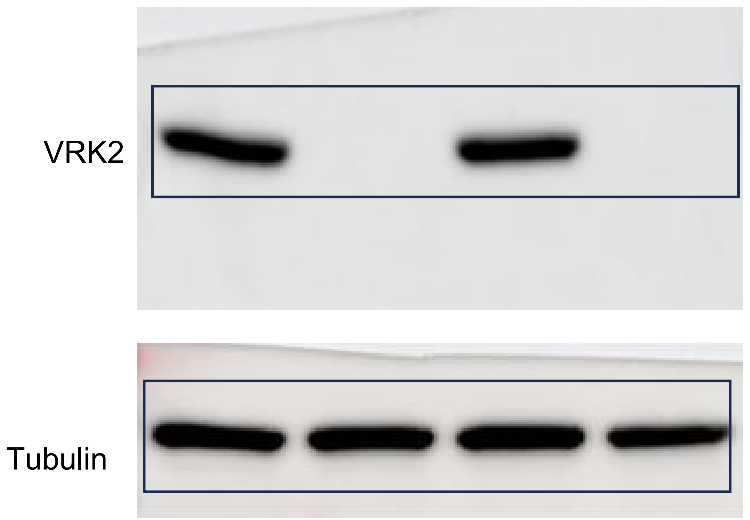

Q

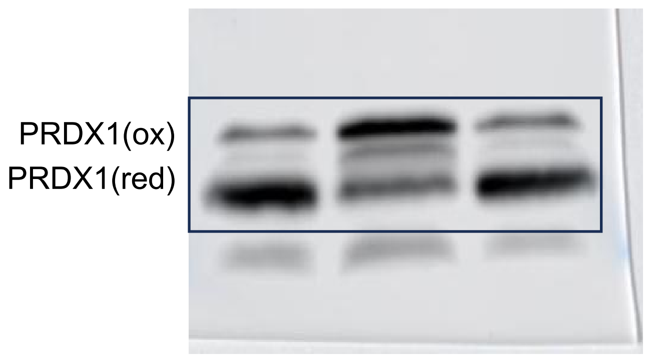

Fig2

B

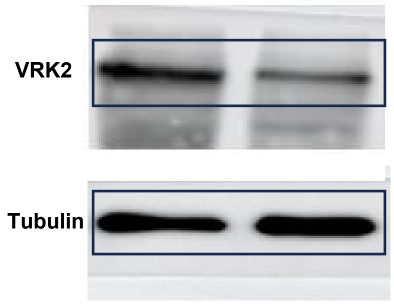

L

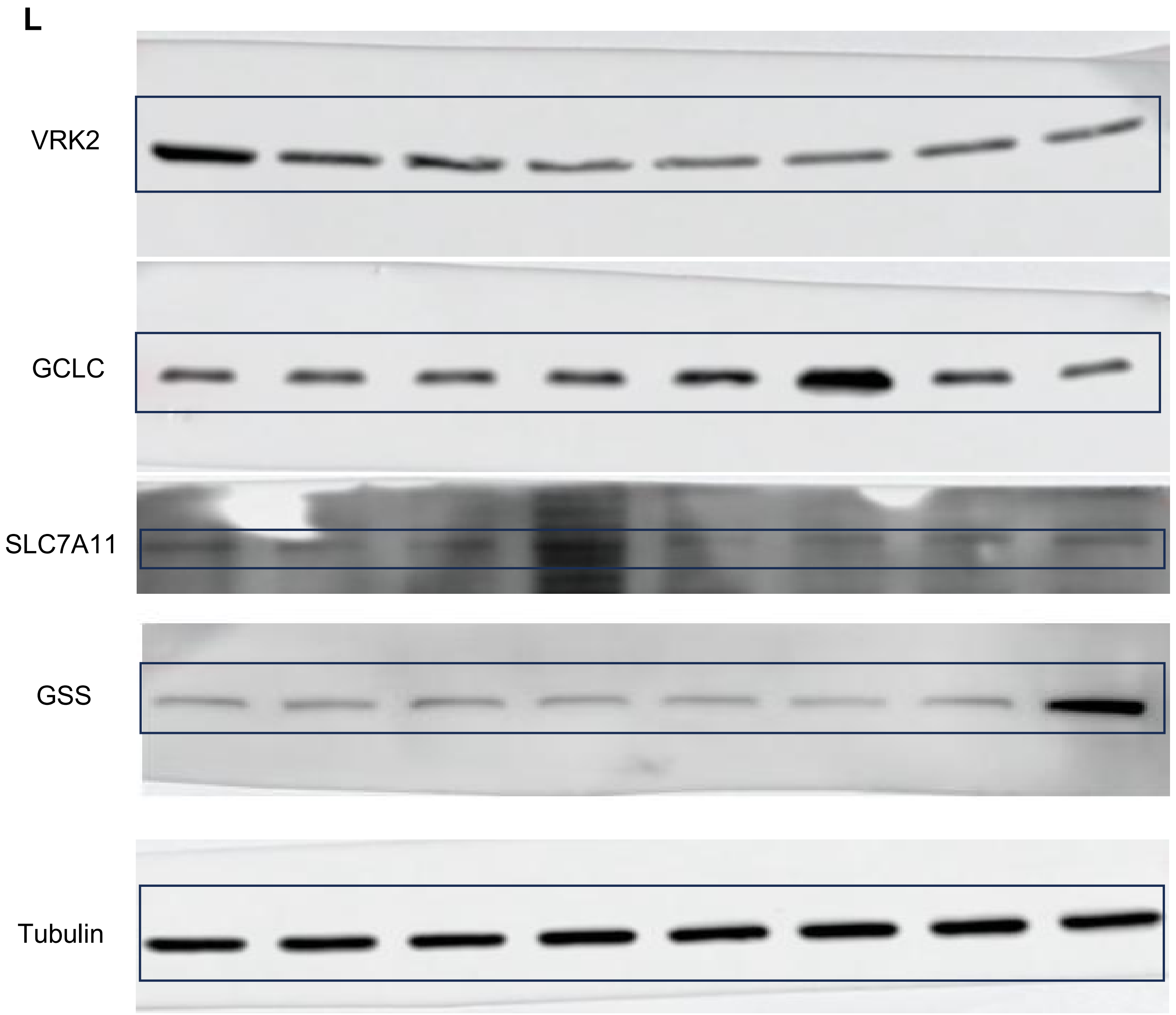

Fig3

B

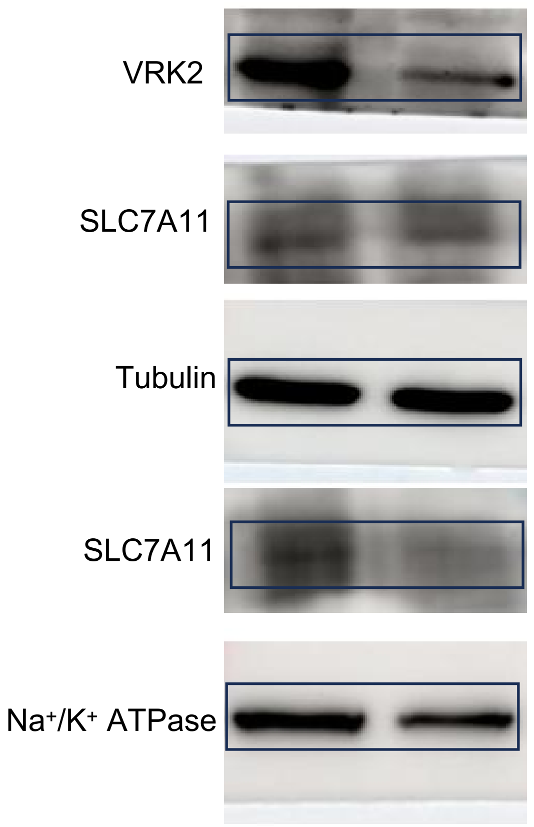

E

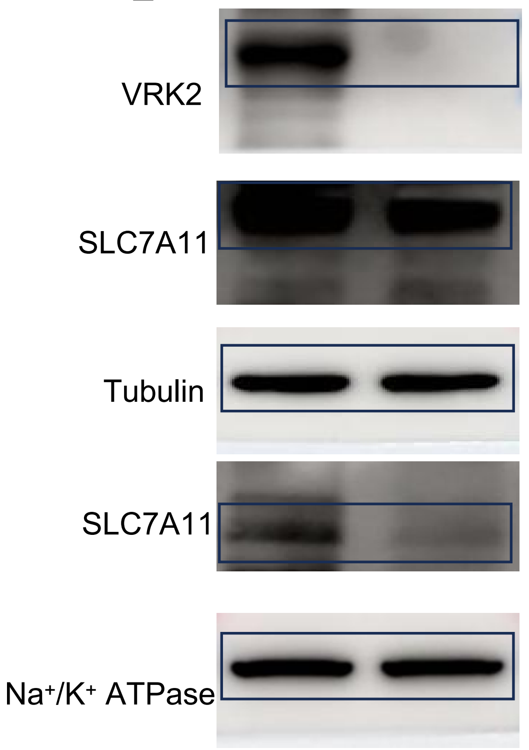

G

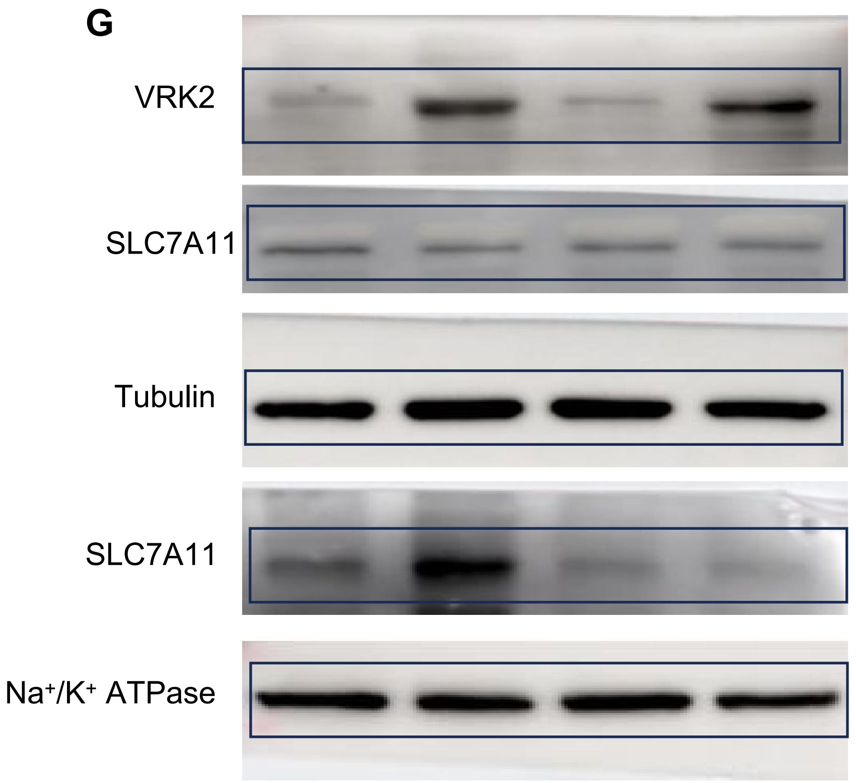

H

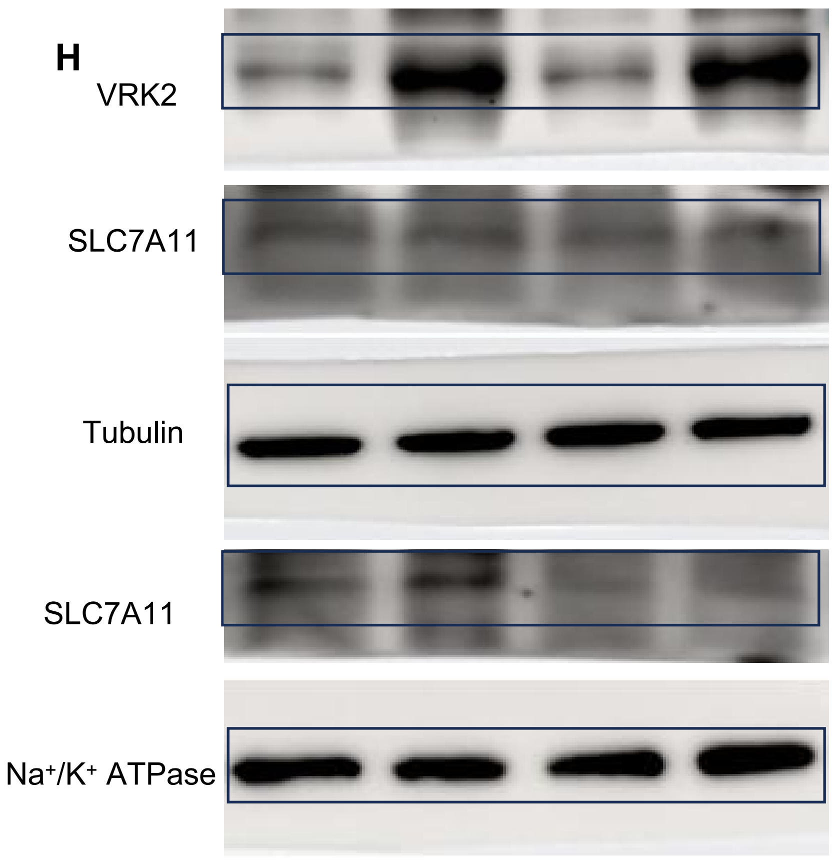

Fig4

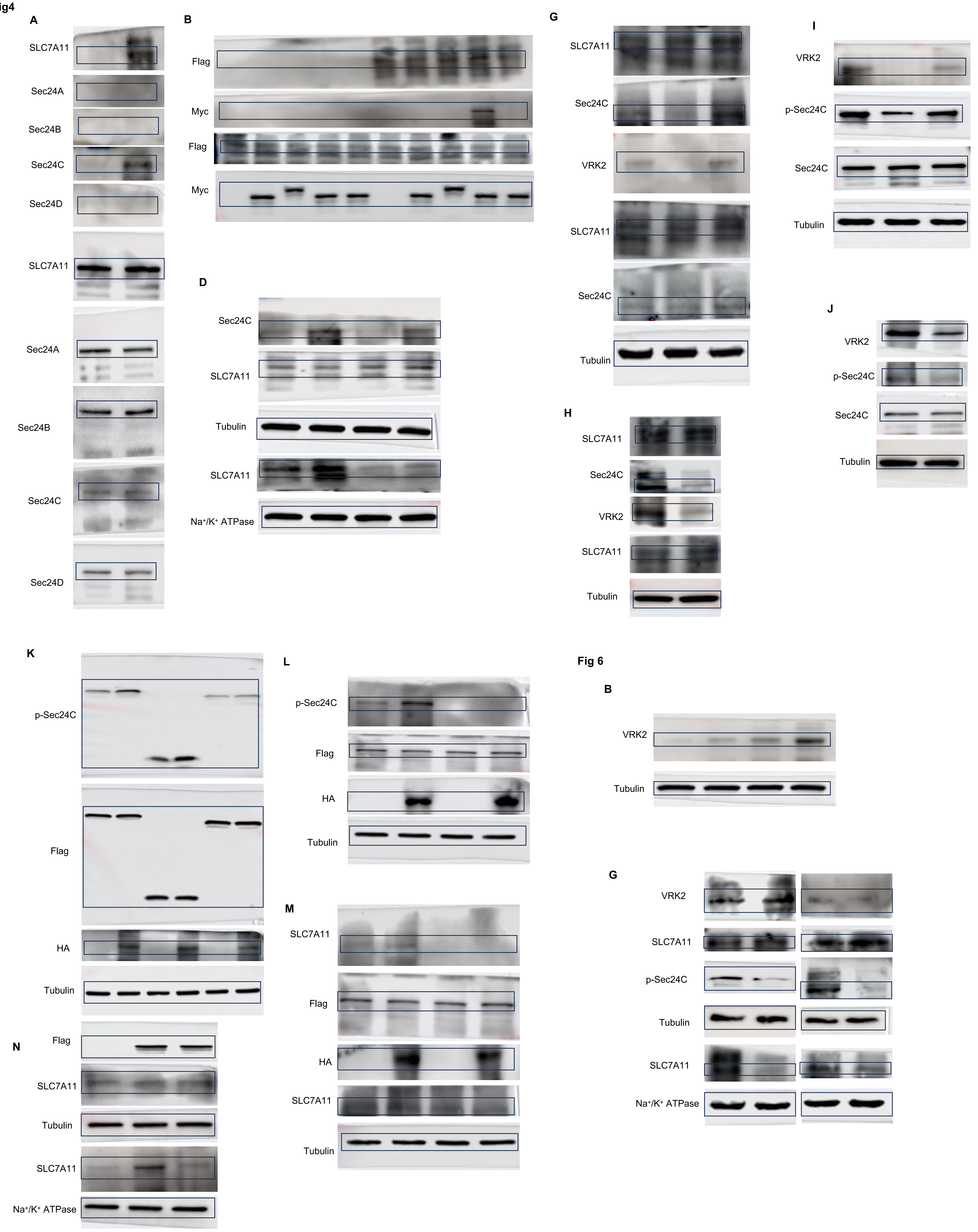

A
SLC7A11
Sec24A
Sec24B
Sec24C
Sec24D
SLC7A11
Sec24A
Sec24B
Sec24C
Sec24D
B
Flag
Myc
Flag
Myc
D
Sec24C
SLC7A11
Tubulin
SLC7A11
Na+/K+ ATPase
G
SLC7A11
Sec24C
VRK2
SLC7A11
Sec24C
Tubulin
H
SLC7A11
Sec24C
VRK2
SLC7A11
Tubulin
I
VRK2
p-Sec24C
Sec24C
Tubulin
J
VRK2
p-Sec24C
Sec24C
Tubulin
K
p-Sec24C
Flag
HA
Tubulin
L
p-Sec24C
Flag
HA
Tubulin
M
SLC7A11
Flag
HA
SLC7A11
Tubulin
N
Flag
SLC7A11
Tubulin
SLC7A11
Na+/K+ ATPase
Fig 6
B
VRK2
Tubulin
G
VRK2
SLC7A11
p-Sec24C
Tubulin
SLC7A11
Na+/K+ ATPase

Supplementary Fig1

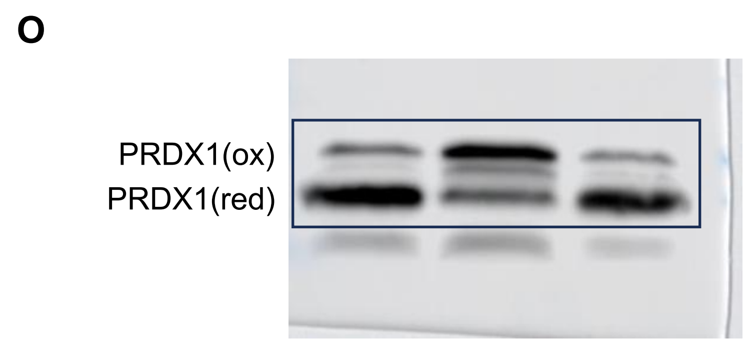

Supplementary Fig2

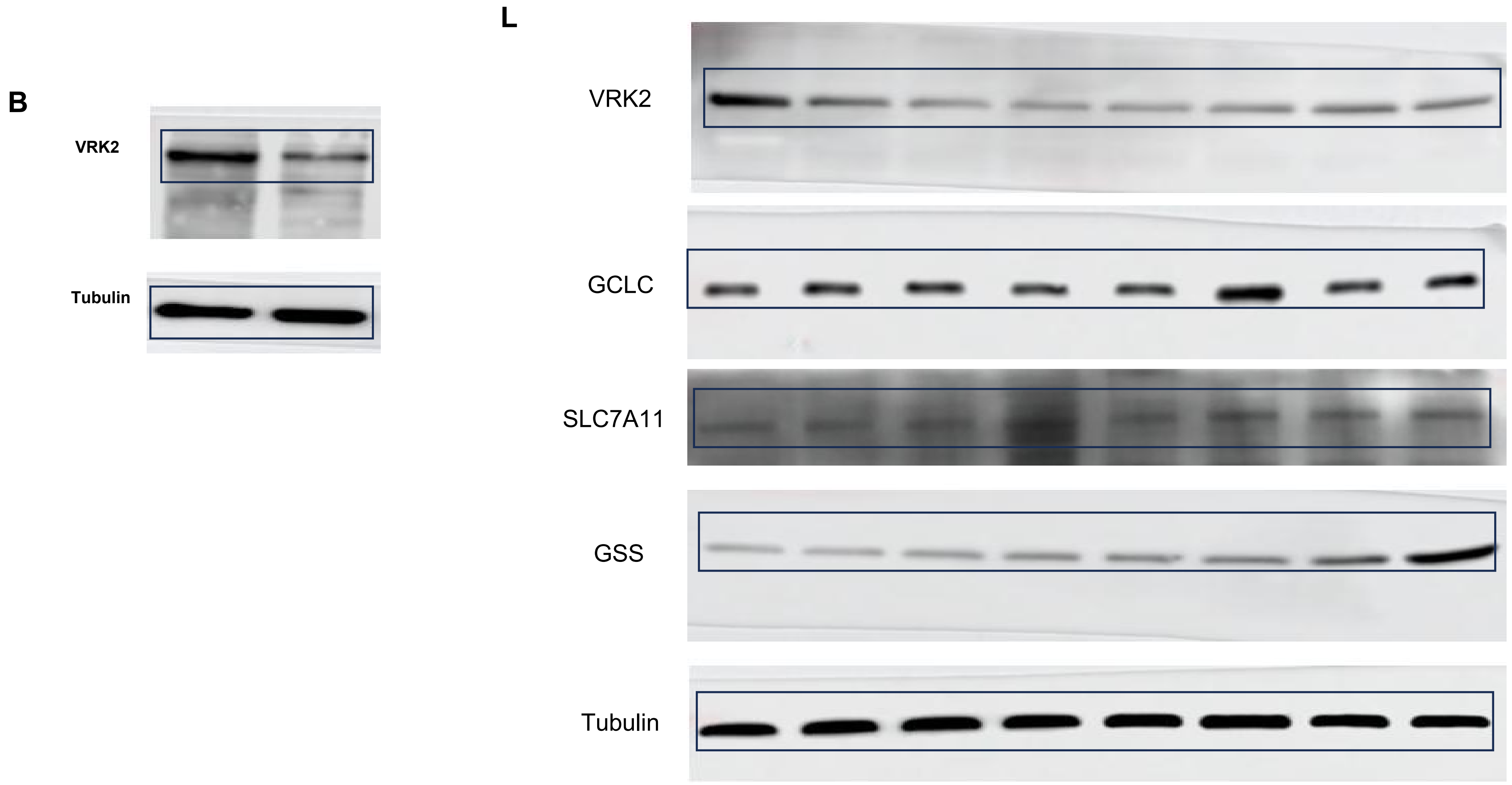

Supplementary Fig3

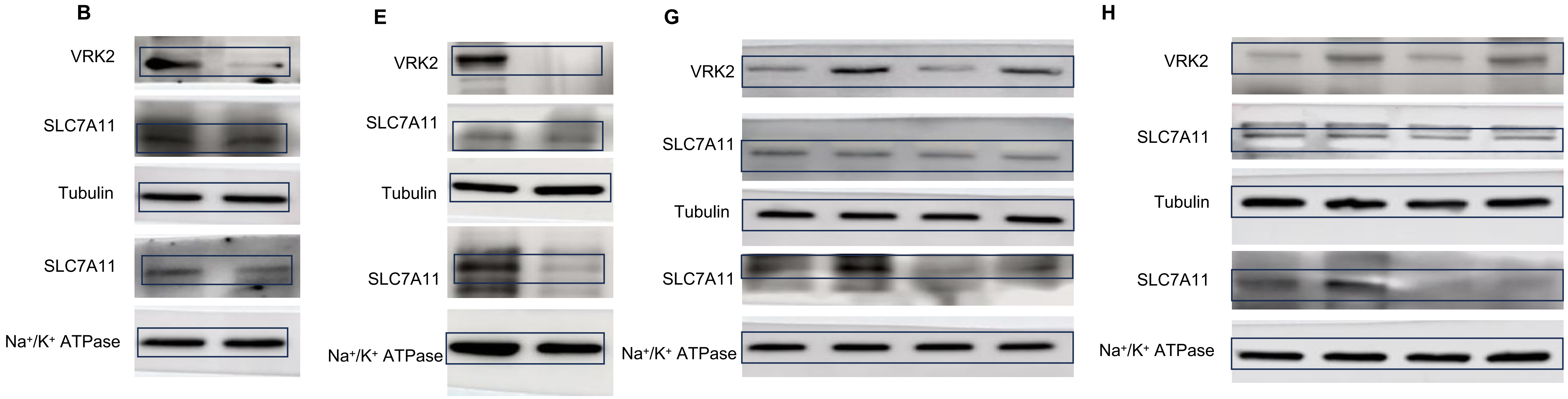

Supplementary Fig4

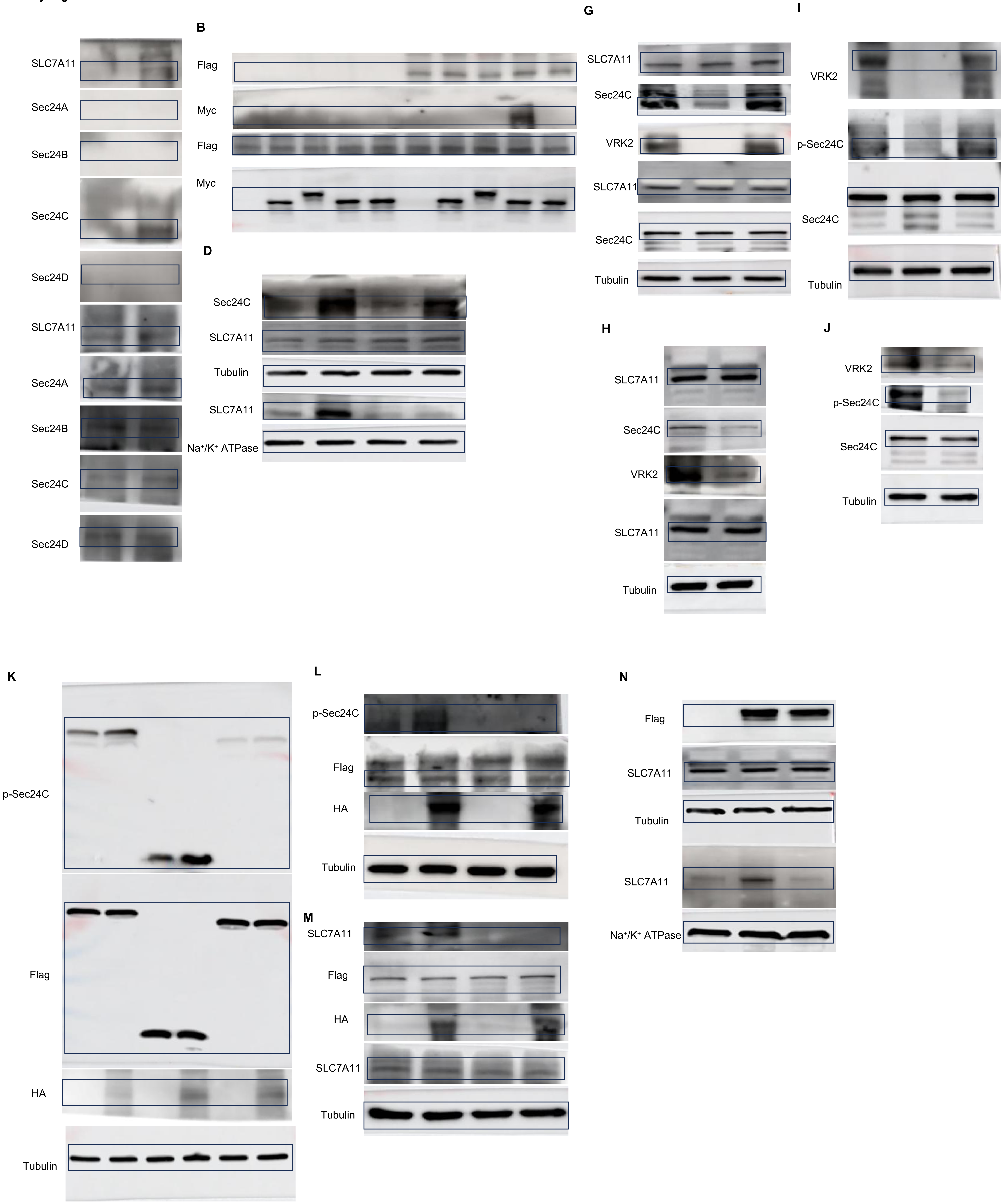

Supplement: Supplementary file 4 — Raw Data [file 41419_2026_8573_MOESM4_ESM.pdf]
